# Thermodynamics and Transport Properties of Heavy Aromatics in a Mixture of DMSO and Toluene Solvents

**DOI:** 10.1021/acsomega.5c08781

**Published:** 2025-11-28

**Authors:** Farid Taherkhani

**Affiliations:** † Departments of Thermodynamics and Thermal Process Engineering Brandenburg University of Technology, Cottbus 03046, Germany; ‡ Energiespeicher-und Energiewandlersysteme, 38871 Brandenburgische Technische Universität, Cottbus−Senftenberg, Cottbus 03046, Germany

## Abstract

To investigate the self-diffusion coefficient of colloidal heavy aromatic aggregates in DMSO and toluene, molecular dynamics simulations were carried out using both the newly developed potential model and the Optimized Potentials for Liquid Simulations force field. The diffusion behavior of the heavy aromatic core in these mixed solvents, as predicted by both models, exhibits a subdiffusive regime. Moreover, the density of heavy aromatic molecules obtained from simulations with the new potential model agrees well with available experimental data. The simulations further indicate that the size of heavy aromatic aggregation nanoclusters in the mixed solvent is approximately 2.7 nm, consistent with experimental observations.

## Graphical abstract

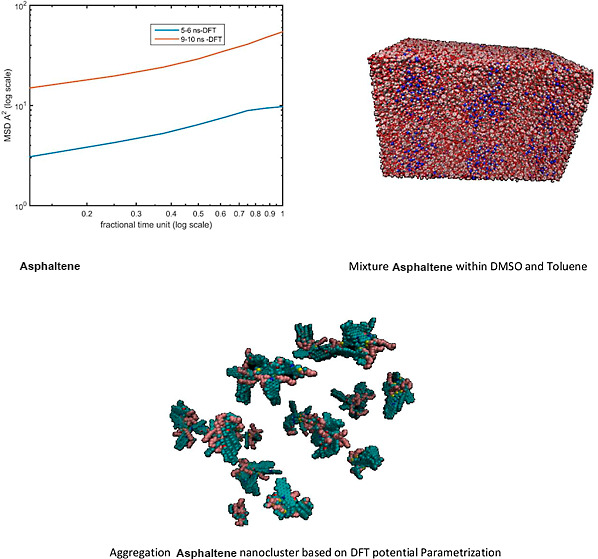

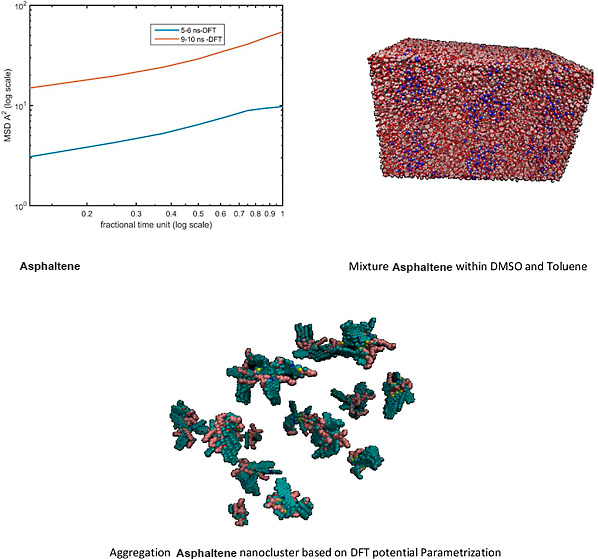

## 1 Introduction

The petroleum industry is undergoing a clear shift toward the processing of heavier crude oils and residues, which are enriched in constituents such as heavy aromatics, resins, and nondistillable hydrocarbons.^1^ These heavy fractions present significant challenges for petroleum production and refining, requiring substantial technical efforts and financial resources. Issues such as pipeline fouling, reservoir surface maintenance, and catalyst deactivation by deposits in reactors are among the critical areas that demand attention and investment. Crude oil itself is a highly complex mixture of components, as extensively documented.^2,3^

Extensive research has provided overviews of commercially available model compounds used to design, synthesize, and study the physical and chemical behavior of heavy aromatics.^4^ These species, which typically represent the nonvolatile fractions of crude oil, are insoluble in *n*-alkanes.^5,6^ Although the precise molecular structures of heavy aromatics remain elusive,^7–9^ they are generally considered to consist of polycondensed aromatic ring systems with aliphatic side chains.^10–12^ In addition, they frequently contain heteroatoms such as oxygen, nitrogen, and sulfur, as well as metals including nickel and vanadium.^13^

The states of heavy aromatics in crude oil remain the subject of ongoing debate.^14^ Quantum chemistry studies employing density functional theory (DFT) have been used to investigate both the thermo-oxidative decomposition of model molecules such as Quinolin-65 and the aggregation behavior of heavy aromatics.^15,16^ As colloidal components in crude oil, heavy aromatics exhibit self-association and aggregation influenced by physical parameters such as temperature, pressure, and chemical composition.^17^ Previous studies have utilized molecular dynamics simulations and DFT calculations to probe these aggregation processes.^18^

A variety of experimental techniques have also been employed to explore heavy aromatics aggregation. Photon correlation spectroscopy provides insights into aggregation dynamics, while small-angle neutron scattering (SANS) has proven valuable for studying their structural organization and aggregation in toluene under varying conditions of temperature and concentration.^19,20^ Furthermore, confocal microscopy offers a powerful tool for visualizing aggregation behavior in organic solvent mixtures such as toluene and heptane.^21^ Complementary computational studies using polarizable continuum model (PCM)-based DFT calculations have demonstrated that solvent effects significantly alter both the energy and Gibbs free energy of organic compounds.^22^

Monte Carlo simulations have been applied to investigate the dynamic evolution of the size distribution of heavy aromatics aggregates.^23^ Molecular dynamics simulations have also been used to examine the thermal stability of heavy aromatics in Khalif crude oil.^24^ Using molecular dynamics, the average hydrogen bonding and aggregation numbers of heavy aromatics have been evaluated in the presence and absence of alkylphenols,^25^ and the influence of chain flexibility on cluster structural formation has been explored.^25^ The average cluster size of heavy aromatics has likewise been analyzed through molecular dynamics simulations,^26,27^ while the radial distribution function has been determined to provide insights into their structural organization.^28^ Furthermore, molecular dynamics simulations have been employed to study the structural configuration and binding energy of dimeric heavy aromatics molecules.^29^

Within the field of quantum chemistry, density functional theory (DFT) has been extensively used to calculate the optimal structures of heavy aromatics.^30^ First-principles calculations have been performed to evaluate the charge density associated with the adsorption of monomeric and dimeric heavy aromatics molecules on calcite surfaces.^31^ DFT has also been applied to investigate the role of hydrogen bonding in heavy aromatics aggregation.^32^ In addition, the aggregation behavior of heavy aromatics in toluene–water mixtures has been widely studied.^33,34^ DFT calculations for colloidal metallic nanoparticles, as well as in aqueous environments, have proven useful for predicting the transport and thermodynamic properties of organic molecule mixtures in water.^35–37^

Accurate charge assignment is crucial for reliable molecular dynamics simulations of heavy aromatics in polar solvents. Because the OPLS (optimized potentials for liquid simulations) force field does not incorporate the correct molecular charges, the present study derives accurate charges for heavy aromatic molecules using DFT. This parametrization enables a more faithful description of aggregation phenomena, consistent with experimental observations.

Aggregation processes are of particular importance in precipitation phenomena in salt technology, and the aggregation of Iranian heavy aromatics poses a significant challenge to the petroleum industry. As shown in Figure 1, heavy aromatics are present in Iranian oils and have also been observed in Tatar oils. To address this challenge, this study performs charge parametrization of heavy aromatics via DFT, with the goal of determining their static and dynamic properties.

**Figure 1:**
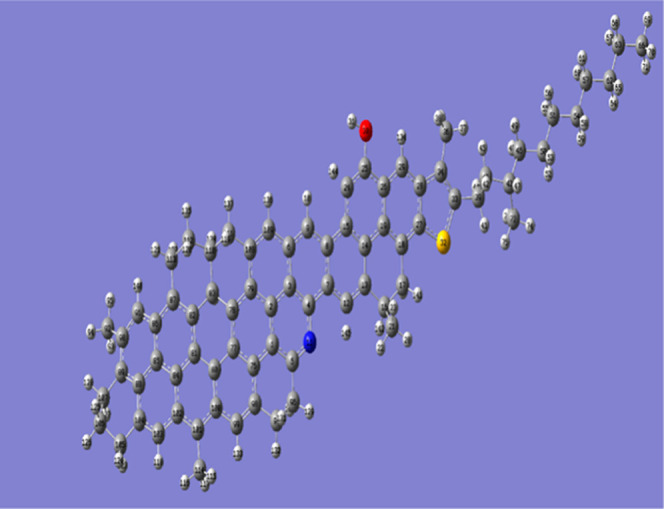
Initial structure for labeled heavy aromatic molecules; S, O, N, C, and H are presented with yellow, red, blue, black, and gray color, respectively.

To further explore aggregation behavior, molecular dynamics simulations were conducted to monitor the time evolution of heavy aromatic cluster sizes. The density of colloidal heavy aromatics in mixed toluene/dimethyl sulfoxide solvents was evaluated, along with the diffusion and aggregation dynamics of the heavy aromatics core as a function of time.

## 2 Computational Method

### 2.1 DFT Calculation Method

In previous literature reports, DFT potential parametrization was conducted for colloidal silver nanoparticles in an ionic liquid. DFT calculations in a solvent have found numerous applications in the design of electrochemical sensors.^36,37^ The optimization of the heavy aromatic structure in both the gas phase and solvents such as dimethyl sulfoxide (DMSO) and toluene was carried out using the Gaussian 16 quantum chemistry package. To calculate certain force field parameters of the heavy aromatics structure, including charge, bond length, and bond angle, the polarizable continuum model (PCM) was employed with the B3LYP method and the 6–31G* basis sets. B3LYP combines Becke’s three-parameter exchange functional with the Lee–Yang–Parr correlation functional.^38,39^

Commonly used split-valence basis sets (6–31G, 6–311G) are usually attributed to the Pople’s basis sets^40,41^

Table 1 presents the results obtained from the DFT calculations using the B3LYP method and 6–31G* basis set, specifically focusing on the point charge of atoms in the heavy aromatics molecules. According to Table 1, the terminal methyl C atom (C 92) in the heavy aromatic structure has a charge value of −0.5422, which arises from its interaction with three hydrogen atoms. It is worth noting that the electronegativity of hydrogen is lower than that of carbon, leading to the carbon atom acquiring a negative charge.

**Table 1:** DFT Potential Parametrization for Point Charge of Atoms and Comparison Point Charge of DFT Calculation and Point Charge of the OPLS Force Field

DFT point charge	OPLS	DFT point charge	OPLS point charge	DFT point charge	OPLS point charge
1 C −0.018451	CA 0.00000	49 H 0.126228	HC 0.06000	97 C-0.359522	CT −0.00500
2 C 0.089413	CA 0.00000	50 C −0.259610	CT −0.12000	98 C 0.162197	CA −0.11500
3 C 0.022754	CA 0.00000	51 C −0.253287	CT −0.12000	99 C −0.345277	CA −0.11500
4 C 0.137853	CA 0.33900	52 H 0.132295	HC 0.06000	100 C 0.136323	CA 0.00000
5 C 0.265141	CA 0.22400	53 H 0.125613	HC 0.06000	101 C −0.008855	CA −0.11500
6 C 0.211847	CA 0.00000	54 C −0.252852	CT −0.12000	102 C 0.139927	CA 0.00000
7 C 0.124011	CA 0.00000	55 H 0.127863	HC 0.06000	103 C −0.335899	CA −0.11500
8 C 0.059035	CA 0.00000	56 H 0.126877	HC 0.06000	104 C 0.154738	CA −0.11500
9 C −0.337870	CA −0.11500	57 C −0.252232	CT −0.12000	105 C −0.349749	CT −0.00500
10 H 0.127039	HA 0.11500	58 H 0.126155	HC 0.06000	106 C −0.268067	CT −0.12000
11 N −0.605052	NB −0.67800	59 H 0.126386	HC 0.06000	107 C −0.350997	CT −0.00500
12 C −0.305175	CA −0.11500	60 H 0.126702	HC 0.06000	108 C 0.143654	CA −0.11500
13 C 0.142122	CA −0.11500	61 H 0.126458	HC 0.06000	109 C −0.314838	CA −0.11500
14 C 0.015748	CA 0.00000	62 C −0.245350	CT −0.12000	110 C −0.084593	CT −0.06000
15 C 0.056178	CA 0.00000	63 C −0.246494	CT −0.12000	111 C −0.361845	CT −0.00500
16 C −0.171663	CT 0.05500	64 H 0.125883	HC 0.06000	112 C −0.264425	CT −0.12000
17 C −0.0360654	CT −0.00500	65 H 0.125705	HC 0.06000	113 C −0.358805	CT −0.00500
18 C 0.075835	CA −0.11500	66 C −0.441243	CT −0.18000	114 C −0.540729	CT −0.06500
19 C 0.012261	CA 0.00000	67 H 0.129770	HC 0.06000	115 H 0.164115	HC 0.06000
20 H 0.140183	HC 0.06000	68 H 0.129610	HC 0.06000	16 H 0.164263	HC 0.06000
21 C −0.438646	CT −0.18000	69 H 0.141064	HC 0.06000	117 H 0.164346	HC 0.06000
22 H 0.151717	HC 0.06000	70 H 0.140724	HC 0.06000	118 H 0.118468	HA 0.11500
23 H 0.157023	HC 0.06000	71 H 0.140845	HC 0.06000	119 H 0.157044	HC 0.06000
24 C −0.283131	CA −0.11500	72 C −0.457294	CT −0.18000	20 H 0.140909	HC 0.06000
25 C 0.306345	CA 0.15000	73 H 0.140836	HC 0.06000	121 H 0.150114	HC 0.06000
26 C 0.112390	CA 0.00000	74 H 0.148215	HC 0.06000	122 H 0.147722	HC 0.06000
27 C −0.251653	C56A 0.00750	75 H 0.144611	HC 0.06000	123 H 0.151133	HC 0.06000
28 C 0.153630	C56B 0.00000	76 C −0.001398	CA 0.00000	124 H 0.144271	HC 0.06000
29 C −0.284996	CA −0.11500	77 C 0.059998	CA 0.00000	125 H 0.145762	HC 0.06000
30 O −0.649960	OHP −0.58500	78 C −0.008905	CA −0.11500	126 H 0.133257	HC 0.06000
31 H 0.409297	HO 0.43500	79 C 0.035411	CA 0.00000	27 H 0.149530	HC 0.06000
32 S 0.191532	SA −0.01500	80 C −0.038594	CA 0.00000	128 H 0.146359	HC 0.06000
33 C −0.186754	C5A −0.10750	81 C 0.021052	CA 0.00000	129 H 0.160206	HC 0.06000
34 C 0.152112	C5B −0.11500	82 C 0.060601	CA −0.11500	130 H 0.162346	HC 0.06000
35 H 0.164881	HC 0.06000	83 C −0.279501	CT 0.17000	131 H 0.163288	HC 0.06000
36 C −0.527926	CT −0.06500	84 C 0.013835	CA 0.00000	132 H 0.145683	HC 0.06000
37 H 0.154711	HC 0.06000	85 C −0.008799	CA 0.00000	133 H 0.121739	HA 0.11500
38 H 0.164454	HC 0.06000	86 C 0.136251	CA 0.00000	134 H 0.148410	HA 0.11500
39 C −0.345336	CT −0.00500	87 C 0.021693	CA −0.11500	135 H 0.132095	HC 0.06000
40 C −0.252479	CT −0.12000	88 C 0.041364	CA 0.00000	136 H 0.117238	HA 0.11500
41 H 0.157606	HC 0.06000	89 C 0.020888	CA −0.11500	137 H 0.141428	HC 0.06000
42 H 0.151130	HC 0.06000	90 C 0.153578	CA −0.11500	138 H 0.127520	HC 0.06000
43 C −0.068583	CT −0.06000	91 C −0.325583	CA −0.11500	139 H 0.184025	HC 0.06000
44 H 0.143027	HC 0.06000	92 C −0.542232	CT −0.06500	140 H 0.120354	HA 0.11500
45 H 0.137646	HC 0.06000	93 H 0.160195	HC 0.06000	141 H 0.153737	HC 0.06000
46 C −0.254184	CT −0.12000	94 H 0.162281	HC 0.06000	142 H 0.134145	HC 0.06000
47 H 0.114589	HC 0.06000	95 H 0.155349	HC 0.06000	143 H 0.163975	HC 0.06000
48 0.126546	HC 0.06000	96 C −0.332900	CT −0.00500	144 H 0.113408	HA 0.11500
				145 H 0.146263	HA 0.11500

### 2.2 Molecular Dynamics for Heavy Aromatic Liquids

Molecular dynamics simulations were performed for a system of 25 heavy aromatic molecules under periodic boundary conditions for 1 ns in the *NPT* ensemble. The optimized structure of a heavy aromatic molecule with a molecular weight of 1032 g/mol, obtained from DFT calculations, is shown in Figure 1.

Partial atomic charges from population analysis are obtained based on Charge Model 5 (CM5). The **CM5 charge model** (Charge Model 5) is a population analysis scheme derived from Hirshfeld charges, designed to better reproduce molecular dipoles and intermolecular interactions.^42,43^

In Figure 1 carbon, hydrogen, nitrogen, sulfur, and oxygen have been shown with gray, white, blue, yellow, and red color, respectively. Initial box size 330 Å × 330 Å × 330 Å for simulation is included for molecular dynamics simulation. Å is used for the Angstrom symbol. The OPLS force field could be used for molecular dynamics simulation of many liquid, organics, and biochemical molecules. Molecular dynamics simulation by using *NPT* ensemble via the OPLS 2005 force field

1 $$\begin{array}{l} E_{non‐bonded}=\sum_{j}q_{i}q_{j}e^{2}/r_{ij}+4\varepsilon_{i,j}\left({\sigma_{i,j}}^{12}/{r_{i,j}}^{12}-{\sigma_{i,j}}^{6}/{r_{i,j}}^{6}\right)\\ E_{bonded}=\sum_{bond}K_{r}{\left(r-r_{eq}\right)}^{2}+\sum_{angles}K_{\theta }{\left(\theta -\theta_{eq}\right)}^{2}+\sum_{dihedral}K_{\phi }\left(1+\cos\left(3\phi \right)\right)\\ E_{total}=E_{bonded}+E_{non‐bonded} \end{array}$$

2 $$\sigma_{i,j}={\left(\sigma_{ii}\sigma_{jj}\right)}^{1/2},\varepsilon_{i,j}={\left(\varepsilon_{ii}\varepsilon_{jj}\right)}^{1/2}$$

lead to volume of simulation box converged and density estimates 1.02 gr/cm^3^ for density of heavy aromatics in bulk structure. First term energy (*E*) in eq 1 shows nonbonded energy of molecules in the system consisting of columbic energy and Lennard-Jones potential between atoms in different molecules. The Second term in eq 1 is *E*_bond_ which has stretching, bending, and dihedral energy term of one molecule in the system. Total energy is summation of bonded and bon-bonded energy, which is presented in eq 1. According to the eq 2, in Lennard-Jones interaction σ_*i*,*j*_, ε_*i*,*j*_ values between two atoms in different molecules are given by geometric mean. Interaction potential between toluene, DMSO, heavy aromatics is taken $\sigma_{i,j}={\left(\sigma_{ii}\sigma_{jj}\right)}^{1/2},\varepsilon_{i,j}={\left(\varepsilon_{ii}\varepsilon_{jj}\right)}^{1/2}$ as a geometrical Lennard-Jones parameter from pure form separately.

There are 210 types of Lennard-Jones interactions for heavy aromatics, which were evaluated in both toluene and DMSO solvents. The detailed Lennard-Jones parameters for solvent–solvent, heavy aromatics–solvent, and heavy aromatics–heavy aromatics interactions are provided in Supporting Information Part C. The parameters employed in the present simulations were taken from the literature.^44–46^ An alternative approach to estimate van der Waals (vdW) interaction energies between heavy aromatics and solvents (e.g., water) is through the OPLS-AA force field, where the total interaction energy is calculated as the sum of nonbonded and bonded terms. According to recent ref 47, vdW interaction energies are specifically derived from the nonbonded interaction terms.

The experimental density of the proposed heavy aromatic structure is estimated to be 1.17 g/cm^3^.^3^ Molecular dynamics simulation results show a deviation of approximately 12% relative to this experimental value. A representative snapshot of the bulk structure of heavy aromatics after 1 ns of simulation, visualized using VMD, is provided. A schematic representation of the molecular dynamics simulation of pure heavy aromatics is shown in Figure S1.

## 3 Results and Discussion

### 3.1 Atomic Charge Distribution Effect on Molecular Dynamics Simulation for Heavy Aromatic Liquids

DFT calculations were performed to determine the charges of the heavy aromatics molecule as input for molecular dynamics (MD) simulations. The simulations were carried out in an initial cubic cell of size (330 × 330 × 330) Å^3^ for 1 ns, modeling bulk heavy aromatics in the liquid phase. The starting temperature was set to 500 K with an annealing step of 50 K. Using force-field parameters derived from DFT, MD simulations were conducted in the *NPT* ensemble. After 1 ns, the calculated density of heavy aromatics was 1.13 g/cm^3^, showing a 3% deviation from the experimental value. A representative snapshot of the bulk structure of heavy aromatics in a DMSO–toluene solvent mixture, obtained after 10 ns of MD simulation with DFT-based potential parametrization, is shown in Figure 2. To investigate the effect of solvent dipole moment on the aggregation size of heavy aromatic components, we selected a high-dipole-moment solvent, DMSO, with a dipole moment of 3.96–4.10 D (1 D = 1 D). The influence of this solvent on the thermodynamic and transport properties of asphaltenes has been examined.

**Figure 2:**
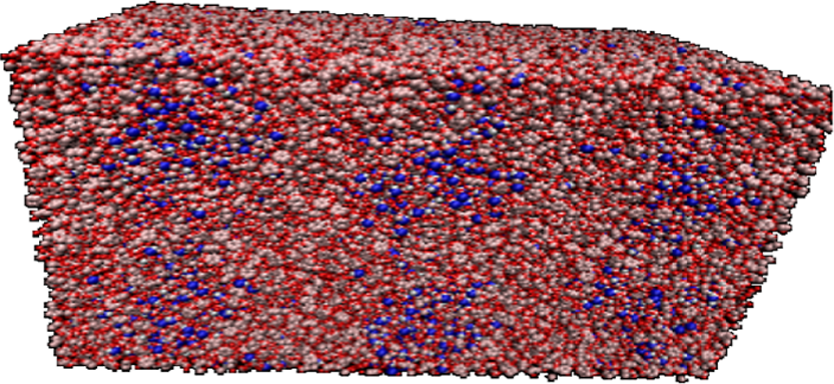
Snapshot of the bulk structure of heavy aromatics in a DMSO–toluene solvent mixture after 10 ns based on DFT potential parametrization.

In Figure 2 yellow, white, blue, green, and red color has been used for sulfur, hydrogen, nitrogen, carbon, and oxygen atoms, respectively.

### 3.2 Diffusion Coefficient of the Heavy Aromatic Core and Tail in OPLS and the DFT Atomic Charge Distribution Effect

Atomistic molecular dynamics simulations were extended to calculate the mean square displacement (MSD) of the core and tail structures of heavy aromatics. These calculations were performed using both the OPLS force field and DFT-based potential parametrization, explicitly accounting for the atomic charge distribution effect. The MSD results obtained with the OPLS force field for aliphatic carbons (CT), aromatic carbons (CA), and nitrogen atoms (N) in the core of heavy aromatics are shown in Figure S1. The simulations revealed that the MSD increased linearly with time. In the results, CT, CA, and N atoms are represented by brown, red, and green color, respectively. The diffusion of aliphatic carbons in the tail was found to be higher than that of aromatic carbons and nitrogen atoms in the core. Moreover, the self-diffusion of nitrogen in the aromatic ring was lower than that of aromatic carbon.

The slopes of the MSD curves were used to evaluate the self-diffusion coefficients. The fitted MSD results for CT, CA, and N yielded values of 3.8 × 10^–11^, 1.8 × 10^–11^, and 1.46 × 10^–11^ m^2^/s, respectively (Figure S2). MSD data were obtained from the HISTORY file of DL_POLY by using the graphical user interface (GUI); however, the GUI did not report uncertainty values.

MSD results based on DFT potential parametrization for nitrogen and aromatic carbons (core) as well as aliphatic carbons (tail) in liquid heavy aromatics are shown in Part S3, represented by dark red, red, and brown color, respectively. MSD for CT in Figure S3 shows tail parts of heavy aromatics liquid. MSD of CA, N in Figure S3 shows heavy aromatic core parts. A direct comparison of MSD values for pure heavy aromatic liquid simulated with OPLS and DFT parametrization is presented in Figure 3. Log–log plots of MSD versus time for N and CA atoms are shown in Figure 4.

**Figure 3:**
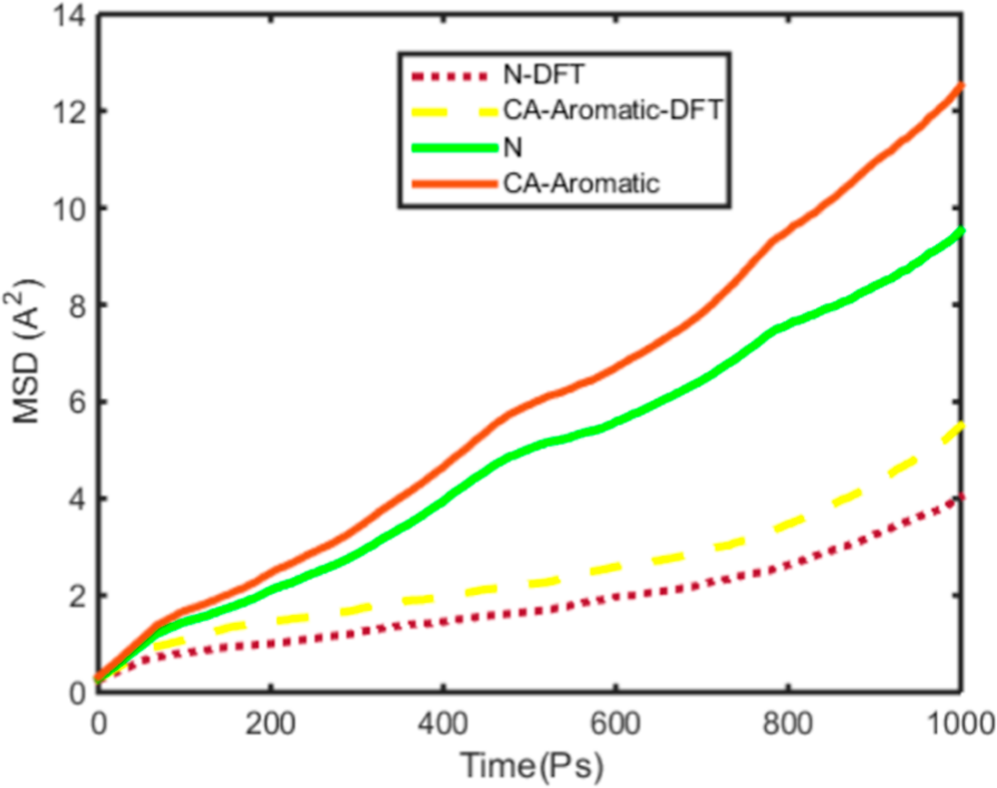
Mean square displacement of the N atom and aromatic carbon in heavy aromatic molecules versus time picosecond with the OPLS force field with solid lines and the DFT parametrization force field for N and carbon aromatics in point line and dash line, respectively.

**Figure 4:**
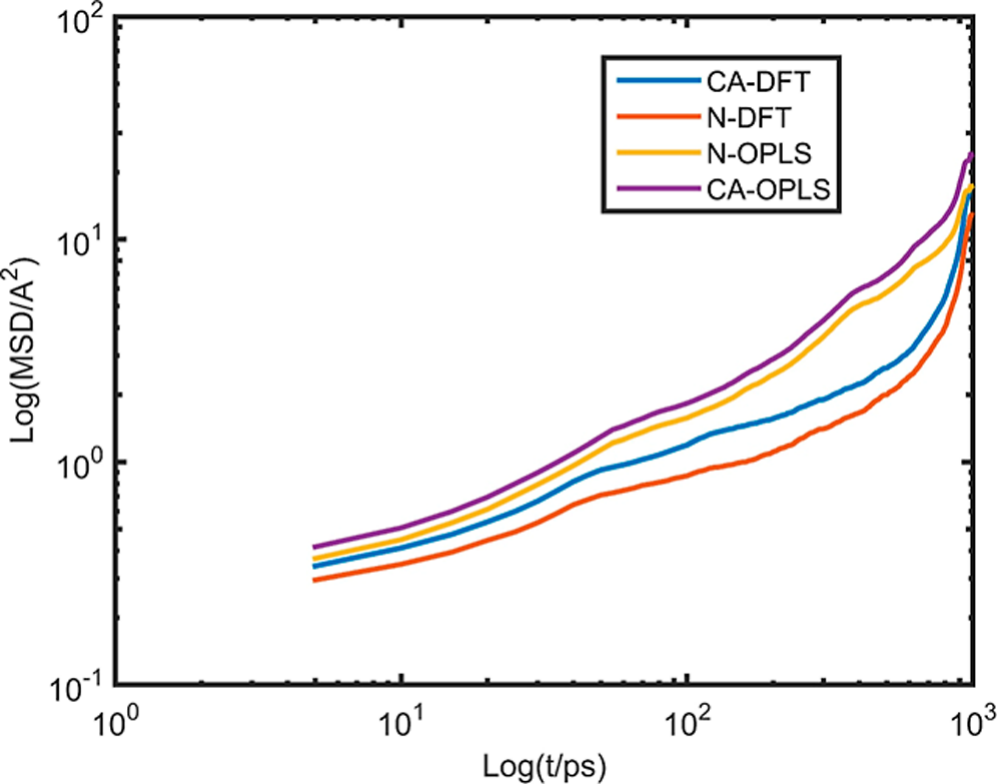
Log (MSD) versus Log (t/ps) for CA and N atoms in heavy aromatics by using DFT potential parametrization with blue and red color, respectively, at 300 K and Log (MSD) versus Log (t/ps) by using the OPLS force field for CA and N atoms in heavy aromatics with purple and yellow color, respectively, at 300 K.

The diffusion regimes for CA and N atoms, as obtained from DFT parametrization, are illustrated in Figures S4 and S5, while the corresponding OPLS results are shown in Figures S6 and S7. Across all cases, the MSD data indicate subdiffusive motion for CA and N atoms, regardless of whether an OPLS or DFT-based potential was employed.

According to Figure 3, the self-diffusion of the core structure of heavy aromatics, when DFT potential parametrization is used, is lower than when using the OPLS force field based on Table 1.

The electrostatic charges obtained from the DFT approach are generally higher than those derived from the OPLS force field. Generally, the difference charge for aliphatic carbon between DFT and OPLS results is more than those for aromatic carbon. Similar values for oxygen charge could be observed in both OPLS and DFT potential models. Consequently, the stronger electrostatic interactions between heavy aromatics and solvent molecules restrict the mobility of the heavy aromatics, resulting in a lower self-diffusion coefficient when using the DFT-based force field compared to that obtained with the OPLS force field.

For simplicity of explanation detail of results, calculation methods, following abbreviations have been used as shown in Table 2.

**Table 2:** Abbreviation Word Which is Used within Text

symbol	abbreviation
MSD	mean square displacement
GUI	graphical user interface
DFT	density functional theory
OPLS	optimized potentials for liquid simulations
CA	aromatic carbons
CT	aliphatic carbons
PCM	polarizable continuum model
DMSO	dimethyl sulfoxide
SANS	small-angle neutron scattering
vdW	van der Waals
RDF	radial distribution function

### 3.3 Radial Distribution in Heavy Aromatics with OPLS and DFT Force Field Parametrization

Molecular dynamics simulations show that incorporating an atomic charge distribution effect through density functional theory (DFT) parametrization does not significantly alter the trend of the radial distribution peak for pure heavy aromatic molecules. This suggests that electrostatic interactions have only a minor influence on the radial distribution function of heavy aromatics in the gas phase. The radial distribution functions of heavy aromatics in the liquid phase, obtained using both the OPLS force field and DFT-based potential parametrization, are presented in Figure S8.

### 3.4 Molecular Dynamics Simulation for Pure Toluene and DMSO Liquid in Bulk Structure

Molecular dynamics simulations in the *NPT* ensemble were performed for pure toluene and DMSO over a duration of 1 ns. The densities of both solvents, calculated using DFT-based potential parametrization, showed excellent agreement with experimental values, with a relative error of only ∼1%.

### 3.5 Molecular Dynamics for Heavy Aromatic Mixture in Toluene and DMSO Solvent

The size and shape of heavy aromatic aggregates are strongly influenced by the solvent. We assume that aggregation occurs primarily within the core structure of the heavy aromatics. Based on the core density of aromatic compounds (1.2 g/cm^3^) and a spherical approximation for heavy aromatic clusters (Figure S9), a cluster of 10 heavy aromatic molecules is estimated to have a diameter of approximately 3 nm. A schematic representation of this 10-molecule cluster is shown in Figure S10, with sulfur, oxygen, nitrogen, and carbon atoms depicted in yellow, red, blue, and black color, respectively.

Similarly, a schematic representation of 530 dimethyl sulfoxide (DMSO) molecules is shown in Figure S11. For the solvent mixture, assuming ideal conditions, the following densities are obtained

3 $$heavy\;aromatics\;density\;\left(D_{heavy\;aromatics}\right)=1.17\;g/{cm}^{3},\;with\;a\;weight\;percent\;of\;5\%$$

4 $$DMSO\;density\;\left(D_{DMSO}\right)=1.1\;g/{cm}^{3},\;with\;a\;weight\;percent\;of\;20\%$$

5 $$toluene\;density\;\left(D_{toluene}\right)=0.867\;g/{cm}^{3},\;with\;a\;weight\;percent\;of\;75\%$$

For the molecular dynamics simulation, a cluster of 10 heavy aromatic molecules was randomly arranged according to the weight percentages defined in eq 3. The initial simulation setup consists of 10 heavy aromatics molecules, 530 DMSO molecules, and 1680 toluene molecules, corresponding to weight fractions of 5%, 20%, and 75%, respectively. The heavy aromatic cluster was placed between the DMSO and toluene solvents, and the structure was replicated using a graphical user interface (GUI) package to prepare the MD simulation. The simulation cell for the mixture system was set to 1172 × 1172 × 1758 Å^3^, with a nonbonded interaction cutoff of 500 Å.

During the simulation, the mixing process and variations in cell size were analyzed by using DFT-based potential parametrization, as shown in Figures S12 and S13. Figures S14 and S15 in the Supporting Information illustrate the arrangement of heavy aromatics, DMSO, and toluene during the mixing process, depicted with different molecular models.

Snapshots of the mixture at 0.8 and 1 ns, along with the corresponding cell size and structure using DFT potential parametrization, are presented in Figure S16A,B, respectively. A cutoff of 3 nm was applied for nonbonded interactions between heavy aromatics, DMSO, and toluene in the mixture system.

### 3.6 MSD for Heavy Aromatic Core in Different Times Based on DFT Potential Parametrization

For calculation of MSD, it is calculated based on the following equation

6 $$MSD=\left\langle {\left(r_{fi}\left(t\right)-r_{fi}\left(0\right)\right)}^{2}\right\rangle$$

which has been used in the previous literature.^48,49^ *r*_f_(*t*) in eq 6 is defined as displacement of atom *i* in heavy aromatic compound from center of mass as a reference at time *t* (*r*_fi_(*t*) = *r*_*i*_(*t*) – *r*_cm_(*t*)). *r*_fi_(0) is the position of the heavy aromatic atom from center of mass as a reference at time zero.

MD simulations were performed to investigate the diffusion of the colloidal heavy aromatic core using DFT-based potential parametrization. The mean square displacement (MSD) of the heavy aromatics core, calculated over the 3–10 ns time interval and plotted against fractional time, is shown in Figure S17 in Supporting Information B. As illustrated in Figure S17, the MSD of the heavy aromatic core decreases with increasing aggregation time.

### 3.7 MSD for Heavy Aromatic Nitrogen in Different Times Based on DFT Potential Parametrization

Molecular dynamics simulations were used to evaluate the mean square displacement (MSD) of nitrogen atoms in heavy aromatics as a function of time. The MSD results for nitrogen during aggregation are presented in Figure S18A (Supporting Information). As shown in Figure S18B, the MSD of nitrogen decreases at longer aggregation times.

The MSD of oxygen atoms in the aromatic core, calculated over the 3–10 ns simulation interval, is presented in Figure S18B. These results indicate that oxygen atoms also exhibit reduced MSD values at longer aggregation times.

### 3.8 Comparison MSD for N and O in Core Parts of Heavy Aromatics

A comparison of MSD values for nitrogen and oxygen atoms in the core of heavy aromatics is presented in Figure S19 (Supporting Information). In this figure, the MSD of oxygen atoms is shown with a dashed line, while that of nitrogen atoms is shown with a solid line. The results indicate that oxygen atoms in the colloidal heavy aromatics core exhibit MSD values higher than those of nitrogen atoms.

Figure S20 (Supporting Information) compares the MSD of the oxygen and carbon atoms in the aromatic core. Here, the MSD of carbon atoms (solid line) is greater than that of oxygen atoms (dashed line).

Finally, the MSD of the heavy aromatic core in a mixture of DMSO and toluene, obtained using both DFT and OPLS parametrizations at 6 and 10 ns, is shown in Figure 5.

**Figure 5:**
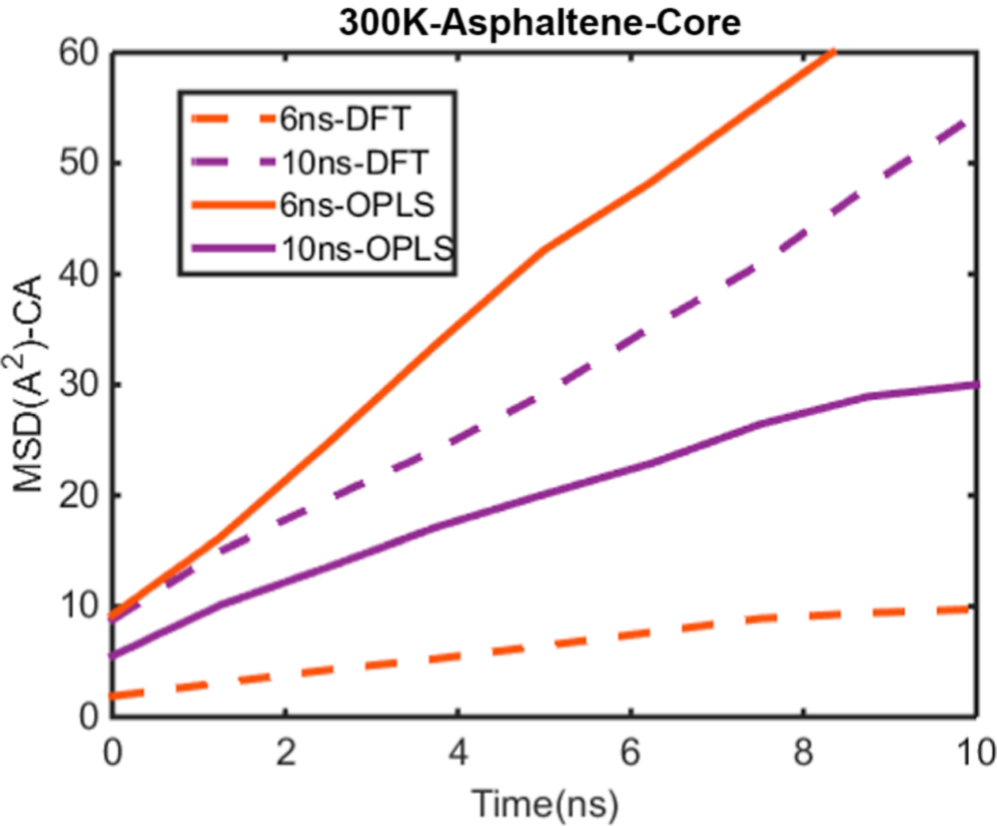
MSD for core aromatic atoms in a mixture of DMSO and toluene by using DFT potential parametrization at different times, 6, 10 ns, as a dash line with red and purple color, respectively. MSD for the OPLS force field at different times, 6, 10 ns, as a solid line with red and purple color, respectively.

Based on Figure 5, the MSD of the heavy aromatics core increases with time when using the OPLS force field, whereas the DFT potential parametrization shows a decrease in MSD with increasing aggregation time. Log–log plots of MSD versus time for the heavy aromatics core at 5–6 ns and 9–10 ns are presented in Figures 6 and 7, respectively, for both OPLS and DFT parametrizations.

**Figure 6:**
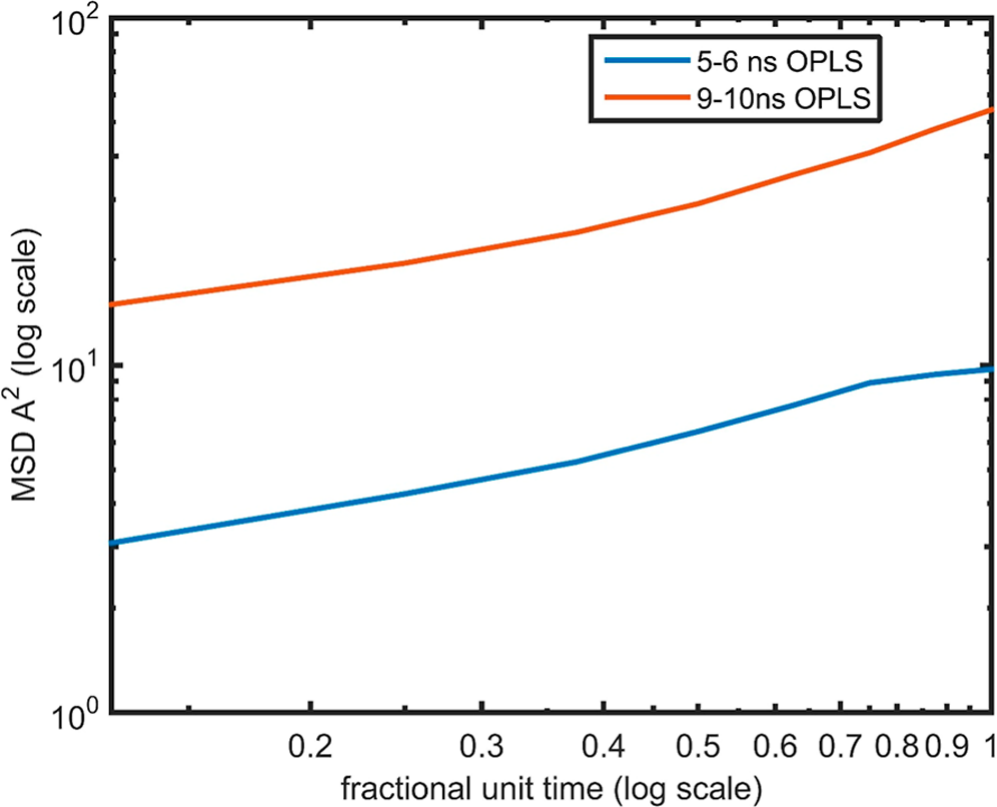
Log–log plot MSD versus fractional time unit for the heavy aromatic core in a mixture of DMSO and toluene solvents via the OPLS force field for simulation time 5–6 ns with blue color and for simulation time 9–10 ns with red color.

**Figure 7:**
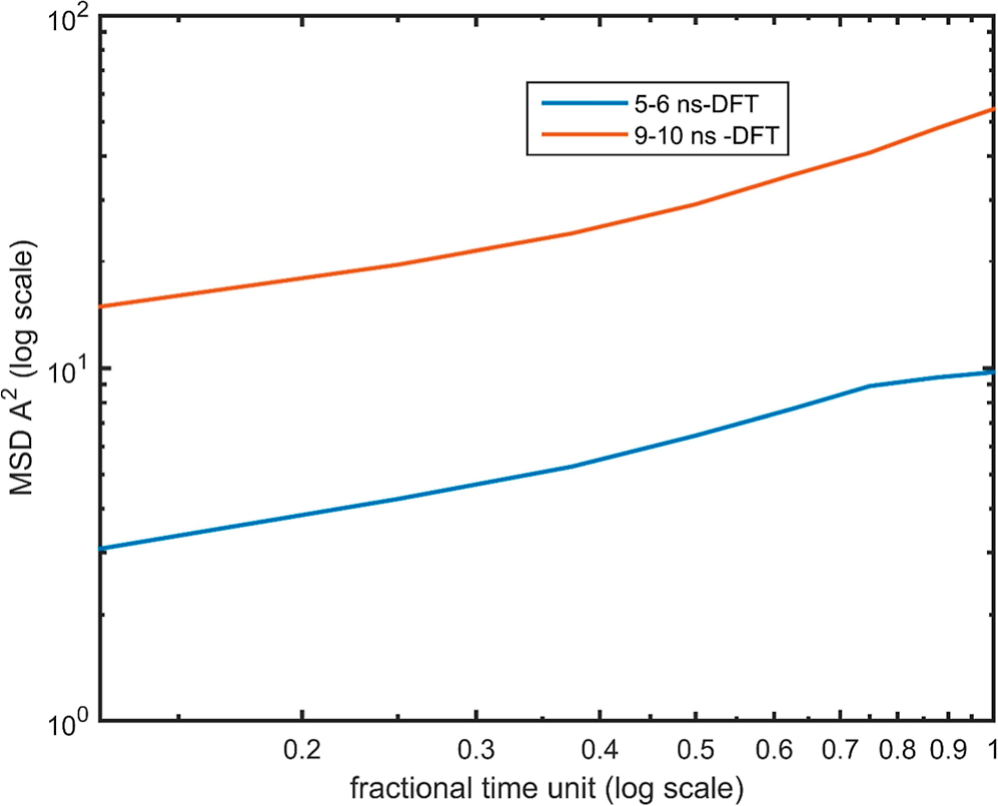
Log–log plot MSD versus fractional time unit for the heavy aromatic core in a mixture of DMSO and toluene solvents via the DFT method for simulation time 5–6 ns with blue color and for simulation time 9–10 ns with red color.

The diffusion regimes of the heavy aromatics core were also analyzed: for 5–6 ns and 9–10 ns using DFT (Figures S21 and S22, respectively) and for the same intervals using OPLS (Figures S23 and S24, respectively). In all cases, the results indicate subdiffusive motion of the heavy aromatic core, regardless of whether the DFT parametrization or OPLS force field was applied.

The MSD results for nitrogen in a mixture solvent (DMSO and toluene) obtained using DFT potential parametrization and the OPLS force field are shown in Figure S25A (Supporting Information), represented by dashed and solid lines, respectively. With DFT parametrization, the MSD of nitrogen in the aromatic core of heavy aromatics decreases with aggregation time, whereas the OPLS force field produces a nonmonotonic trend in the nitrogen self-diffusion coefficient (or MSD) as a function of time. At shorter aggregation times, the MSD values from DFT are higher than those from OPLS; however, at longer simulation times, the OPLS results exceed the DFT values.

Similarly, the MSD results for oxygen atoms in the aromatic core in a mixture of DMSO and toluene solvent are presented in Figure S25B, with dashed and solid lines corresponding to DFT and OPLS, respectively. The DFT parametrization shows a decreasing MSD for oxygen with aggregation time, while the OPLS force field again yields a nonmonotonic behavior.

### 3.9 Temperature Effect on the MSD Result Based on DFT Potential Parametrization

The temperature dependence of the MSD for aromatic carbon atoms in the heavy aromatic core was examined at 300 and 350 K using both OPLS and DFT potential parametrizations. The corresponding MD results are presented in Figure S26A,B for the DFT and OPLS models, respectively. In both cases, an increase in temperature leads to higher MSD values, reflecting the enhanced mobility of aromatic carbon atoms.

### 3.10 Radial Distribution Function for the Heavy Aromatic Core in Different Times by Using DFT Potential Parametrization

Figure S27 in Supporting Information shows the results of molecular dynamics simulations of the radial distribution function (RDF), based on DFT potential parametrization, for carbon–carbon interactions within the heavy aromatic core at 350 K. Figure S28 in Supporting Information presents the RDF between aromatic carbon atoms (CA) in the core and aliphatic carbon atoms (CT) in the side chains at two simulation times, 2 and 10 ns. The results indicate that aggregation time has little effect on CA–CT correlations. In addition, Figure 8 displays the RDF for hydrogen-bonding interactions between oxygen (O) atoms in DMSO solvent and hydrogen (H) atoms in the phenol group of heavy aromatics.

**Figure 8:**
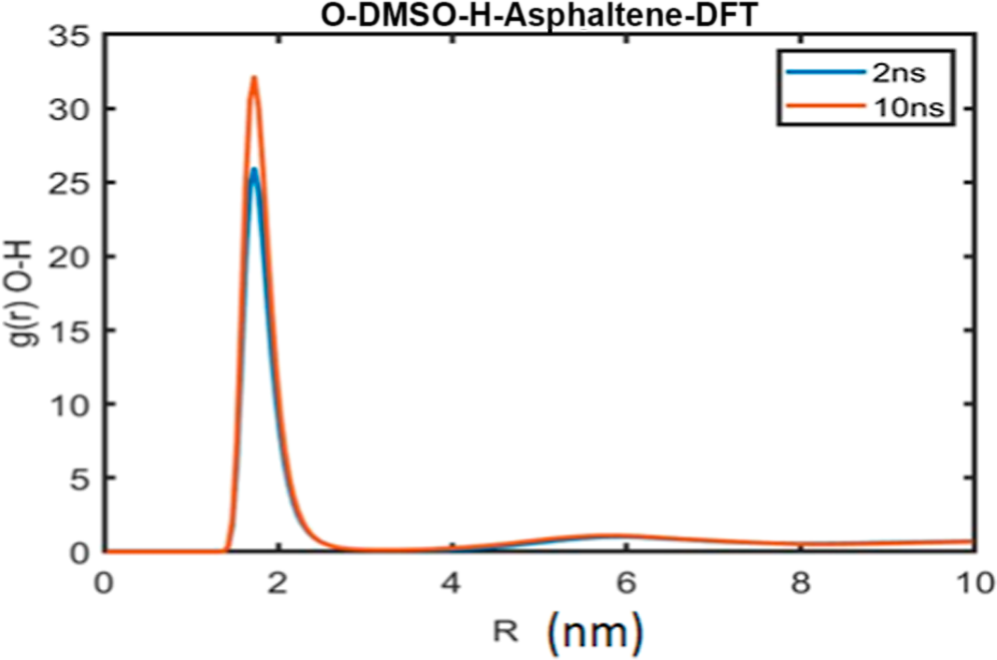
Result of radial distribution function versus distance for hydrogen bonding between O in DMSO solvent and the H atom in the phenol group of heavy aromatics at 2 ns with blue color and at 10 ns with red color.

The figure demonstrates the presence of hydrogen bonding between oxygen atoms in the DMSO solvent and hydrogen atoms in the phenol group of heavy aromatics. The radial distribution function (RDF) peaks of the O–H interactions increase in intensity as aggregation time progresses, while the peak position remains unchanged. The RDF data were obtained from the DL_POLY output file; however, uncertainties are not reported in this output.

Previous studies have shown that heavy aromatics possess a dipole moment, and that solvent effects significantly influence aggregation size due to this dipole moment.^50^ To further investigate heavy aromatic aggregation, molecular dynamics simulations were extended to 100 ns. All solvent molecules (DMSO and toluene) were removed from the mixture solution to observe clear cluster formation of heavy aromatic molecules during the aggregation process. The structural shape of heavy aromatics strongly affects cluster size, and the molecular structure shown in Figure 1 was selected to ensure accurate aggregation size predictions. Our molecular dynamics simulations of cluster size aggregation are consistent with experimental results. Recent studies also highlight the importance of molecular structure on heavy aromatic aggregation.^51^ Furthermore, electrostatic and hydrogen-bonding interactions play a crucial role in determining aggregation behavior.^52–54^

Figures 9 and 10 present the results of cluster formation of heavy aromatic molecules using DFT potential parametrization and the OPLS force field, respectively, over the 10–100 ns time range.

**Figure 9:**
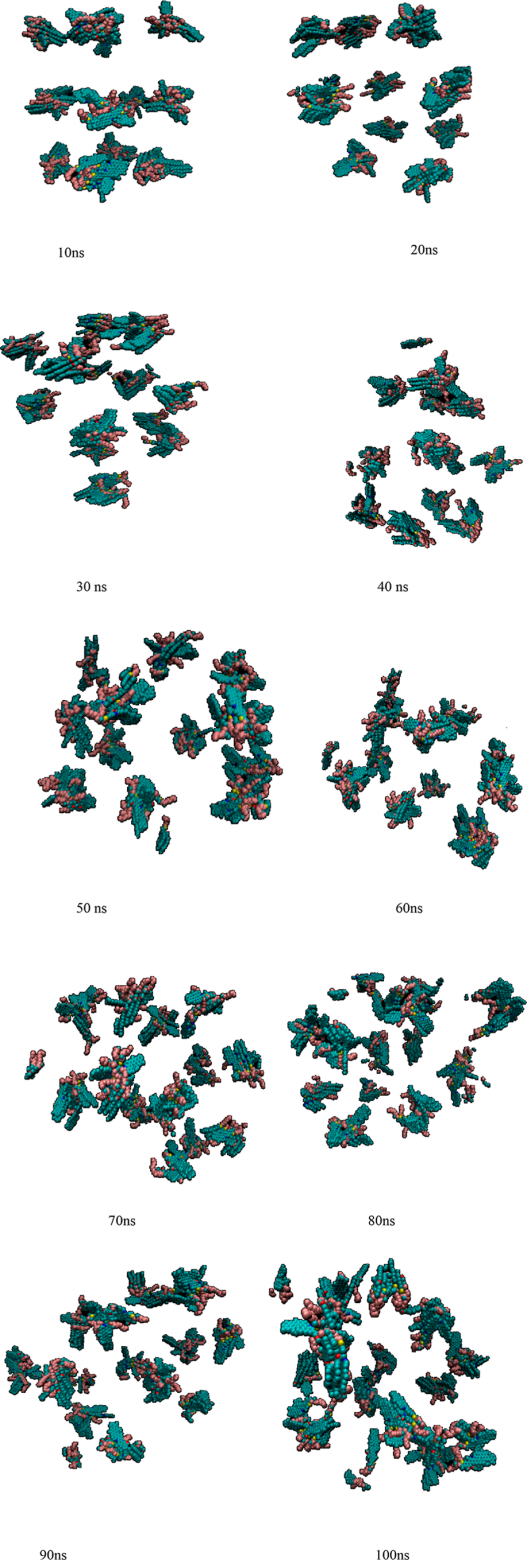
Aggregation in nanocluster formation of heavy aromatic molecules by using DFT potential parametrization during a simulation time of 10–100 ns.

**Figure 10:**
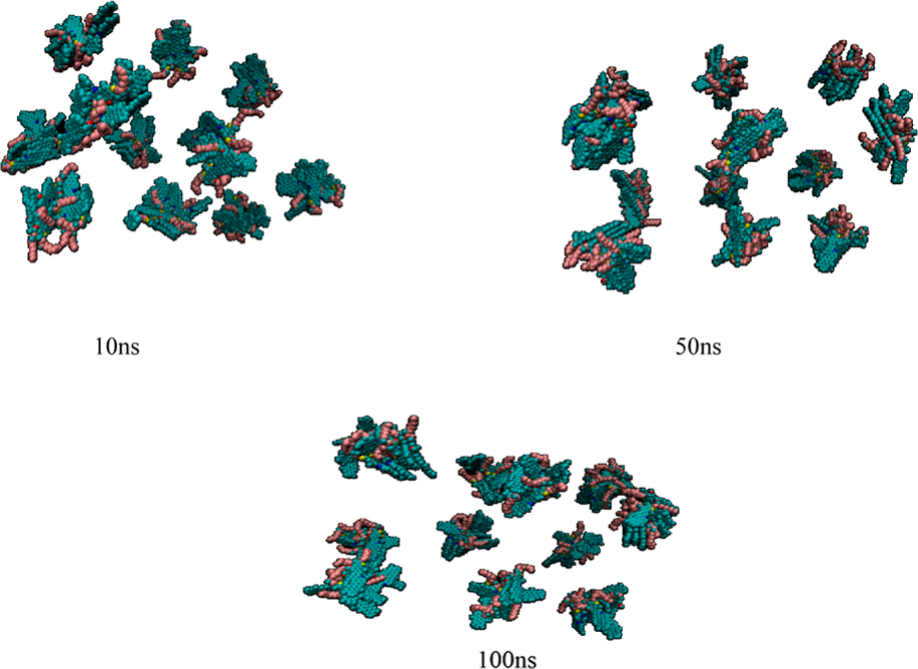
Same as Figure 9 for aggregation in nanocluster formation of heavy aromatics during time 10–100 ns by using the OPLS force field.

The DFT potential parametrization results show that clusters begin to form at the early stages of the simulation and grow larger as time progresses, with the heaviest aromatic molecules tending to aggregate. In contrast, the OPLS force field produces nanoclusters of larger size at shorter simulation times, exhibiting an asymmetric size distribution. However, at longer time scales, the clusters generated with OPLS tend to converge toward similar sizes. For calculation of aggregation of heavy aromatics compounds in DMSO and toluene, a Fortran program has been developed and the Fortran program has been used to analyze trajectory output files of molecular dynamics. As the first step, solvent molecules are separated from the solution. For the second step, the trajectory has been analyzed based on the rest of the molecules, which are heavy aromatic compounds in the aggregation form to get the size of aggregation.

A spherical approximation was employed to estimate cluster sizes within the mixed-solvent system.^55^ In this approach, the center of mass of the heavy aromatic core was first calculated from the DL_POLY history file, and the maximum distance of any heavy aromatic core atom from this center of mass was taken as the cluster radius, using a Fortran-based calculation.^55^ The cluster sizes were also verified graphically by using VMD, showing good agreement with the spherical approximation method.

Based on the MD snapshot shown in Figure 9, the largest cluster size formed during heavy aromatic aggregation with DFT parametrization is approximately 2.7 nm, which agrees well with experimental observations.^56^ In comparison, the maximum cluster size obtained with the OPLS force field is about 2.1 nm, showing a notable deviation from the experimental results. Previous studies have reported that force fields developed via DFT methods generally provide better predictions of transport properties in colloidal solutions.^57^

Figures 9 and 10 both demonstrate that heavy aromatic aggregation increases over time. The shape of nanoclusters strongly influences their thermodynamic and transport properties, with the analysis revealing that most nanoclusters formed during the simulations exhibit amorphous structures rather than perfect spherical shapes. Amorphous terminology here means aggregation structure does not have any symmetry element. It is better to mention that current aggregation structure for heavy aromatic compounds does not have special symmetry element. Owing to this fact, aggregation structure of heavy aromatics has C1 symmetry. There is not Bravais lattice for current structure of aggregate nanocluster for heavy aromatic compounds in mixture solvents. The Fortran program has been developed to specify geometries of metal nanocluster. Analysis of Geometry structure based on our previous Fortran code shows^55,58^ one C1 symmetry and no Bravais lattice crystal symmetry structure for the shapes of aggregation in heavy aromatic compounds. The previous literature shows that the alternative method for calculation aggregate shapes of molecular structure is based on principal moments of inertia.^59–61^

The MD results further indicate that aggregate sizes vary within the range of 1 to 3 nm over the 1–100 ns simulation window.

### 3.11 Molecular Dynamics Simulation for Heavy Aromatics, Pure Solvent, and Mixture of Heavy Aromatics in Solvents

Molecular dynamics (MD) simulations in the *NPT* ensemble were performed to investigate the thermodynamic properties of the pure heavy aromatics. These simulations employed the all-atom OPLS-2005 force field, which was also used to optimize pure toluene and dimethyl sulfoxide (DMSO), as well as mixtures of heavy aromatics with these solvents.

Both static and dynamic properties of heavy aromatics were analyzed by using the OPLS-2005 force field. The simulations were carried out using the DL_POLY 4.09 package^62,63^ in the *NPT* ensemble at room temperature. A time step of 1 fs was used. For pure heavy aromatics, MD simulations were run for 1 ns (corresponding to 10^6^ time steps), with an equilibration period of 500,000 steps. The Nosé–Hoover thermostat/barostat algorithm implemented in DL_POLY 4.09 controlled the temperature and pressure, with relaxation times of 0.1 ps for the thermostat and 2.0 ps for the barostat.

For the heavy aromatic mixture, extensive MD simulations were initially conducted in the *NPT* ensemble at 1 atm and 500 K. An annealing procedure was applied, followed by stepwise cooling in 50 K increments until the system’s total energy converged. Subsequent simulations were performed over the temperature range of 150–370 K to investigate the thermodynamic behavior of the mixture.

The density of heavy aromatics in the mixture, calculated by using both the DFT potential parametrization and the OPLS force field, is presented in Figure 11. As shown in Figure 11, the density exhibits a nonmonotonic behavior during aggregation in the mixture.

**Figure 11:**
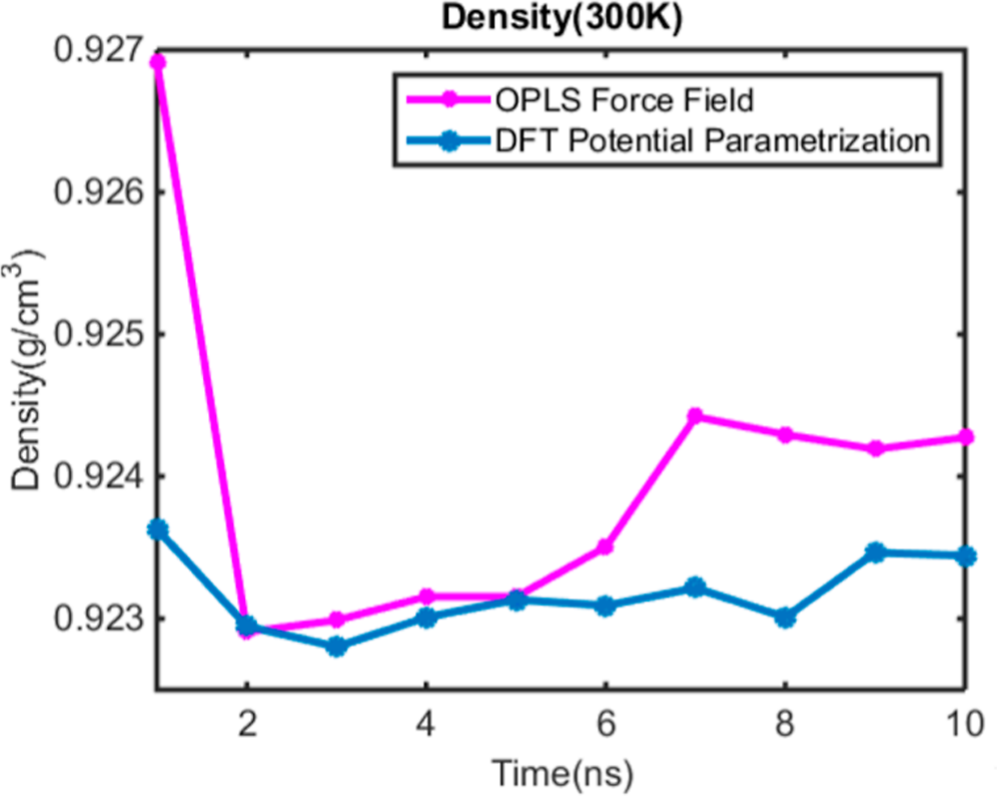
Density of heavy aromatics versus nano simulation time based on DFT new potential with blue color and the OPLS force field with red color.

## 4 Conclusion

Molecular dynamics simulations were conducted to investigate the structure of pure heavy aromatics by using both OPLS and DFT potential parametrizations. The DFT-based parametrization provided a more accurate estimation of heavy aromatic density and aggregation size compared with the OPLS force field.

The simulations were further extended to study the behavior of heavy aromatic mixtures in DMSO and toluene solvents. Results from the DFT parametrization revealed a nonmonotonic variation in mixture density over time, which remained consistently lower than the density predicted using the OPLS force field.

Self-diffusion coefficients of carbon atoms in the core of heavy aromatics within the DMSO mixture were also evaluated. Using the DFT parametrization, the self-diffusion coefficient decreased over time, whereas with the OPLS force field it increased monotonically over the 1–100 ns time range.

Finally, the radial distribution function between oxygen atoms in DMSO and hydrogen atoms in heavy aromatics showed an increase with the aggregation time (Figure 8), indicating enhanced hydrogen bonding between heavy aromatics and DMSO molecules as aggregation progresses.

## Supplementary Material



## Notes

Diffusion coefficient and log (MSD) of core and tail parts of heavy hydrocarbon compounds based on the new developed potential model via the DFT and OPLS force field, radial distribution between two carbon cores in heavy aromatic compounds, schematics representation for mixing of heavy aromatic compounds and DMSO and toluene solvents during the aggregation process, MSD in different fraction times and temperatures for tail and core parts of heavy aromatic compounds in mixture solvents via the new DFT potential and OPLS force field, and radial distribution function for heavy aromatic compounds in mixture solvent (PDF)
