# Supplementary material for: Thermodynamics and Transport Properties of Heavy Aromatics in a Mixture of DMSO and Toluene Solvents
Source: ACS Omega. 2025 Nov 28;10(48):59458–68. doi: 10.1021/acsomega.5c08781 (PMC12771216; doi:10.1021/acsomega.5c08781)
Supplement: Supplementary file 1 [file ao5c08781_si_001.pdf]

Thermodynamics and Transport Properties of Heavy Aromatics in Mixture DMSO and Toluene Solvents

Farid Taherkhani[1,2*]

[1]*Departments of Thermodynamics and Thermal Process Engineering Brandenburg University of Technology, Cottbus, 03046 Germany*

[2] *Energiespeicher-und Energiewandlersysteme, Brandenburgische Technische Universität, Cottbus–Senftenberg, Germany*

[*]Corresponding Author:

taherkha@b-tu.de, faridtaherkhani@gmail.com

Fig.S1.

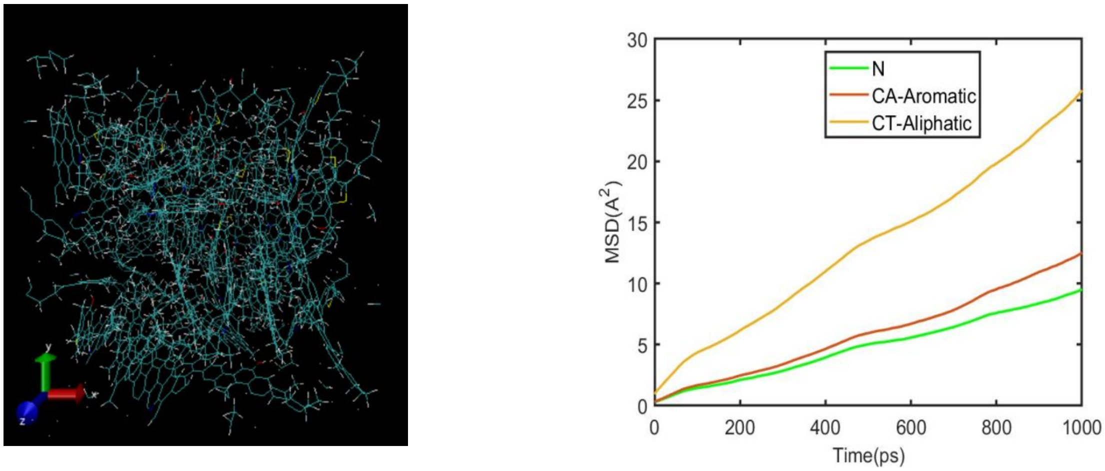

(A) (B)

Fig.S1.A. Shematics presentation of optimized Asphaltene after 1 nanosecond via OPLS force field

Fig.S1.B. MSD result by application of OPLS force field for aliphatic and aromatic carbon and N atom in Asphaltene core as a function of time

Fig.S2

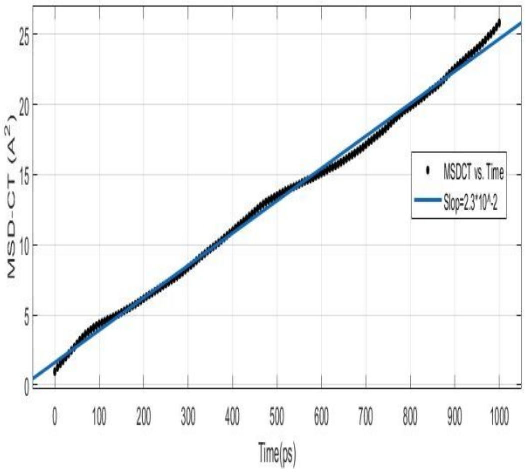

(A)

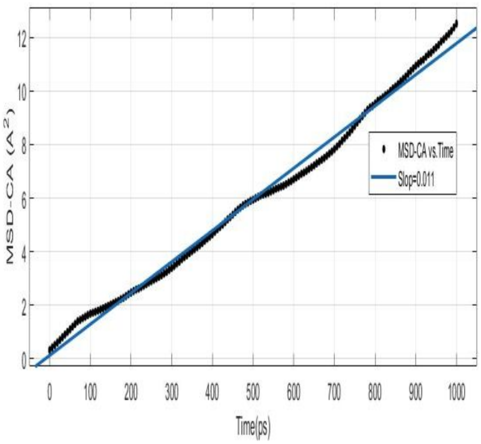

(B)

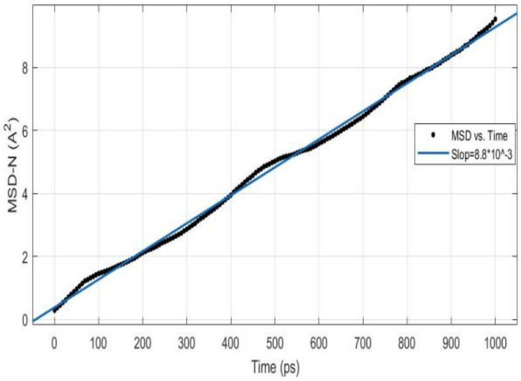

(C)

FigS2.Results of the MSD fitting with one-line shapes for A) CT , B) CA , C) N are presented Fig.S2

Fig.S3

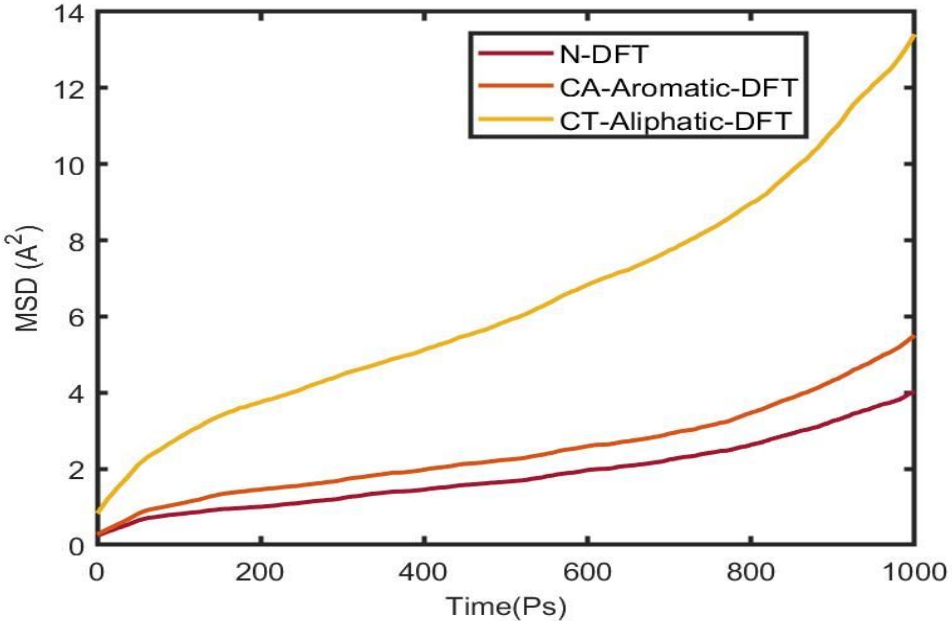

FigS3. Result of MSD via Molecular dynamics simulation based on DFT potential parametrization, for nitrogen and carbon in core structure and aliphatic carbon in tail structure for Asphaltene liquid

Log of MSD for heavy aromatic core versus log t has been shown at FigS4 Result of Fig.4 shows one linear relationship for MSD versus time. M parameter at Fig.S4 shows slop of Log MSD versus log (t) for core atom carbon in heavy aromatic component.

Fig.S4

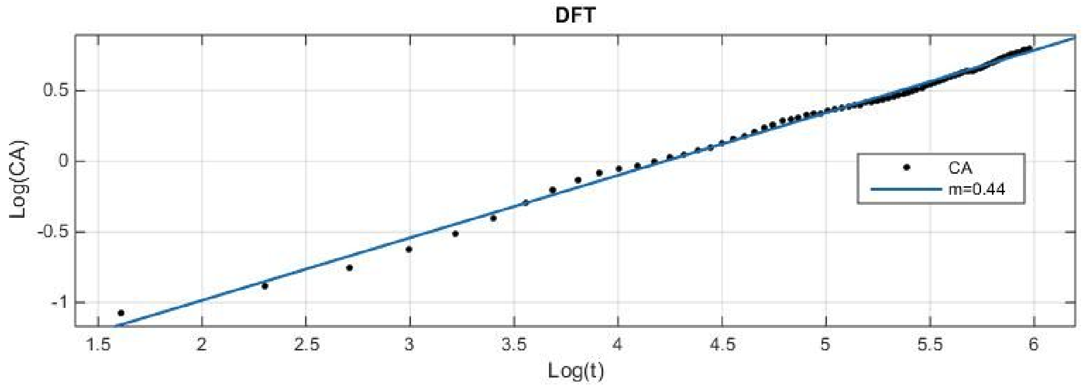

Also Log of MSD for heavy aromatic core versus log t has been shown at FigS5. Result of Fig.S5 shows one linear relationship for MSD versus time. M parameter at Fig.S5 shows slop of Log MSD versus log (t) for Nitrogen atom in core parts in heavy aromatic component.

Fig.S5

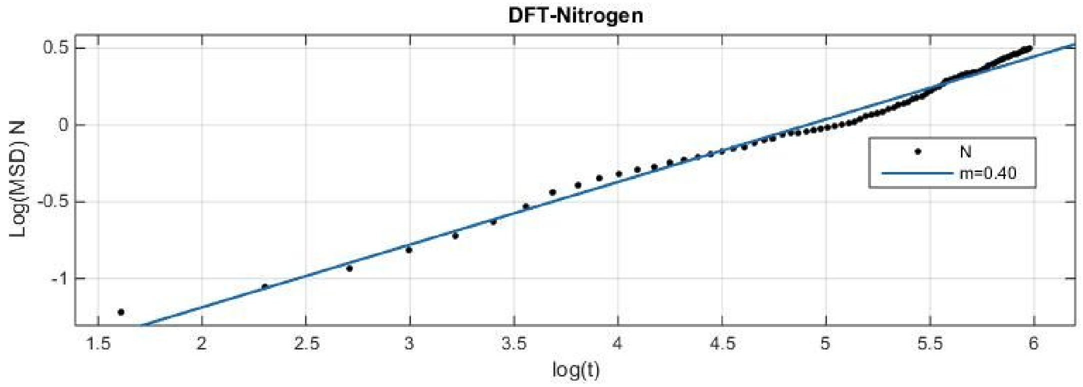

MSD result for carbon and nitrogen in core of heavy aromatic compounds versus log t (time) by using OPLS force field has been presented at Fig.S6 and Fig.S7 respectively. Based on Fig.S6 and Fig.S7, MSD shows one linear scale for log (CA) versus Log (t) and slop for carbon atom in core CA is more than N atom.

Fig.S6

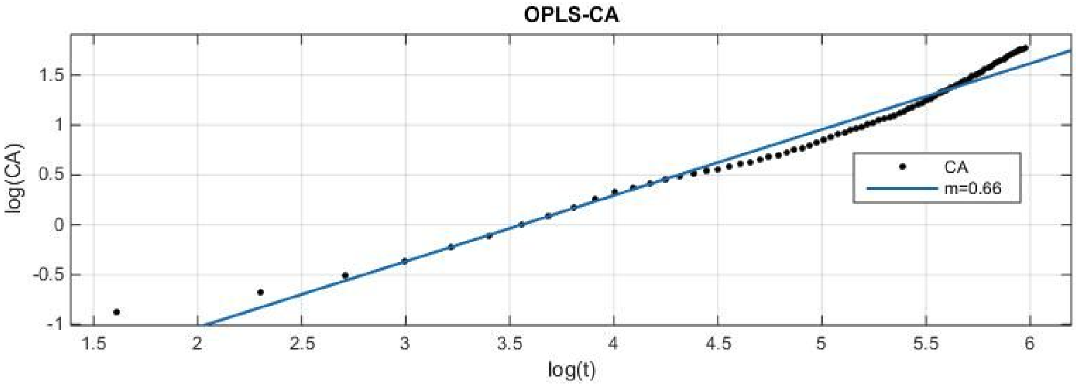

Fig.S7

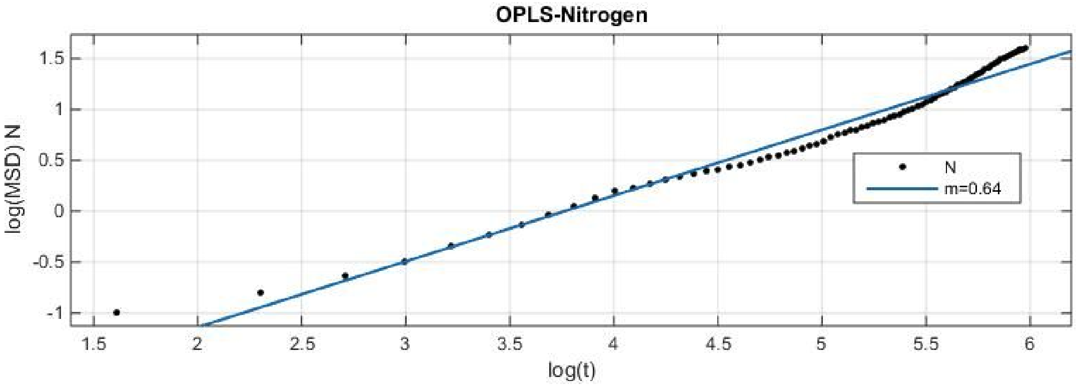

Fig.S8

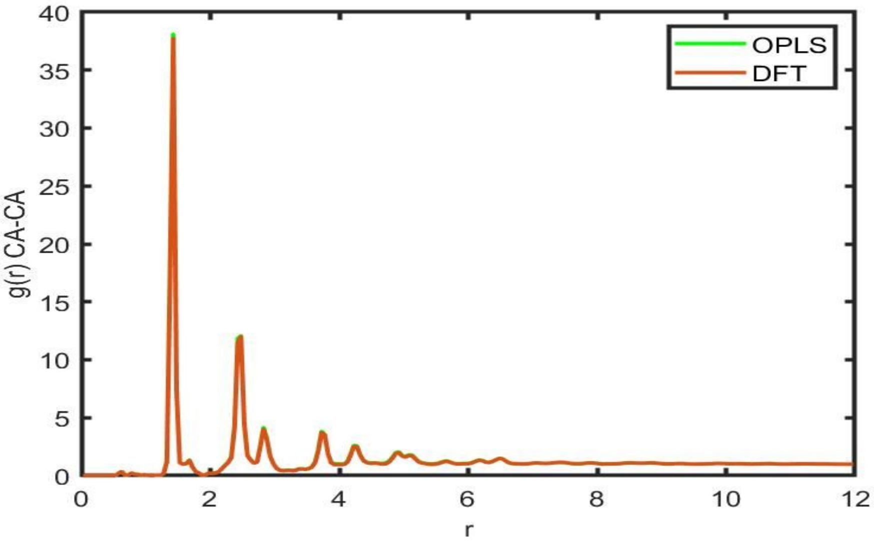

Fig.S8. MD result for radial distribution function for Asphaltene liquid with OPLS force field and DFT potential parametrization

Also Log(MSD) versus Log(t) for heavy aromatic core (Asphalten core) in two different time 5-6, 9-10 ns via DFT potential shows one linear relationship.

Fig.S9

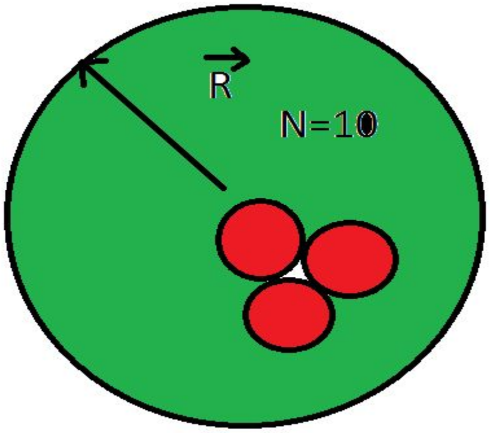

Fig.S9. Ten Asphaltene molecule is included for formation of Asphaltene cluster with 3 nm size

Fig.S10.

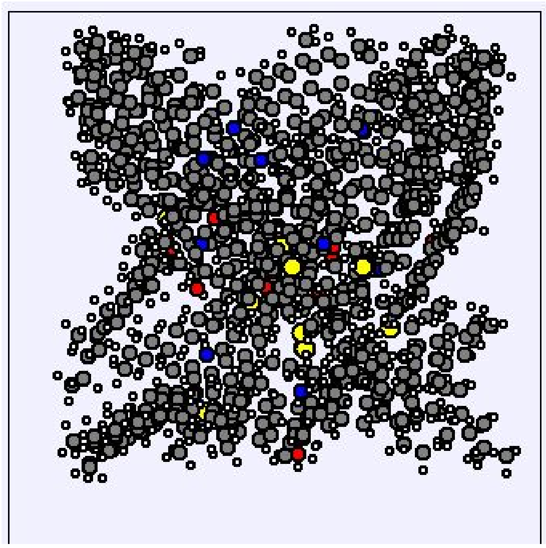

FigS10.Schematic representation of a 10 Asphaltene cluster

Fig.S11

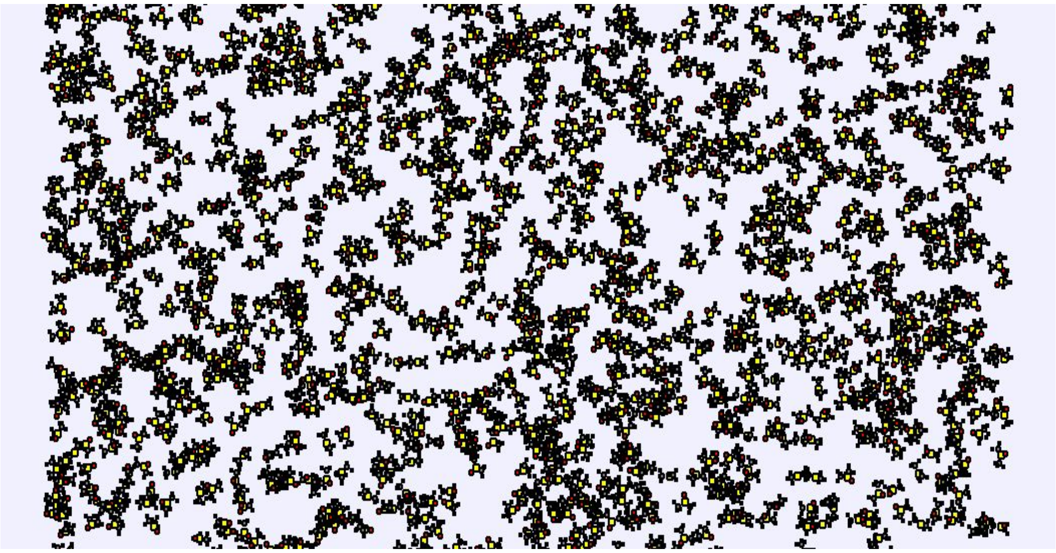

Fig.S11. Schematic representation of 530 molecules of dimethyl sulfoxide (DMSO) solvent

For performing MD simulation, we have used 10 pure heavy aromatics and 530 molecules of dimethyl sulfoxide (DMSO) solvent and 1680 toluene molecules. Heavy aromatics and solvent molecule such as DMSO should be optimized first before mixing. Schematics presentation of optimized 10 molecules of heavy aromatics and 530 molecules of DMSO solvent has been presented at Fig S10. and Fig.S11 respectively.

Snapshot for mixing process of 5%Asphalt, 20 %DMSO, 75%Toluen via MD simulation with including time dependent the volume of mixture with time has been presented at Fig.S4.S5,S6.

Fig.S12

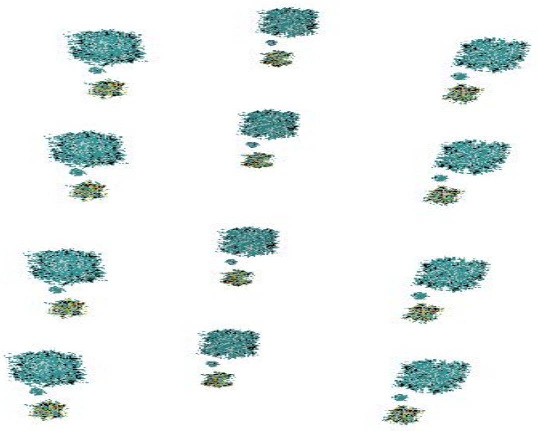

Fig.S12.MD simulation setup with Initial Cell Size (1172*1172*1758 A^3)

Weight (5%Asphalt, 20 %DMSO, 75%Toluen)

Fig.S13

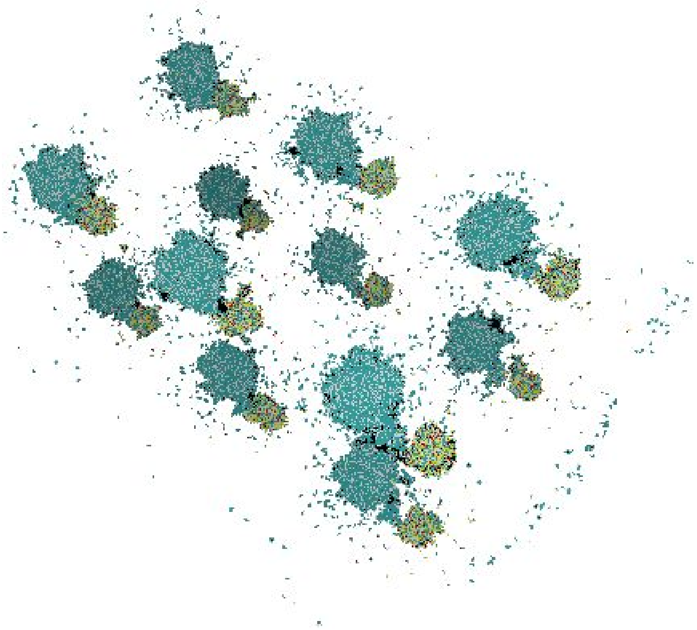

Fig.S13. MD Snapshot with Cell size (1026*1026*1540) A^3

Weight (5%Asphalt, 20 %DMSO, 75%Toluen)

Fig.S14

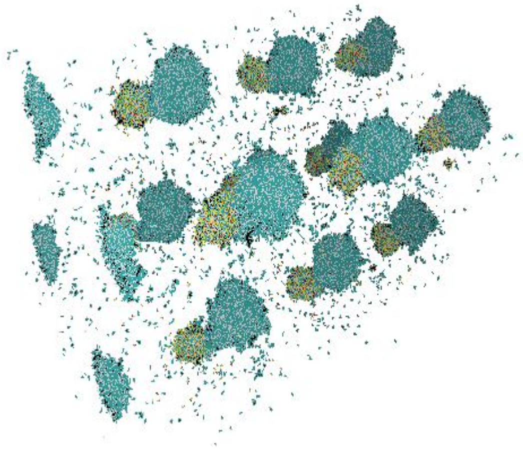

Fig.S14.MD snapshot with (470*470*705)A^3

Weight (5%Asphalt, 20 %DMSO, 75%Toluen)

Fig.S15

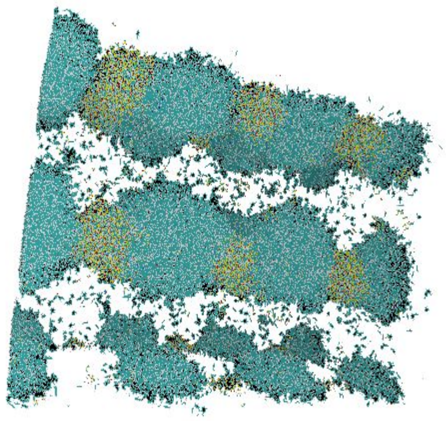

(A)

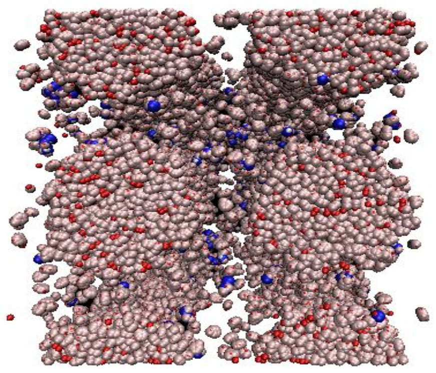

(B)

Cell size (324*324*407)A^3 Weight (5%Asphalt, 20 %DMSO, 75%Toluen)

Fig.S15.A . Combining Asphaltene , DMSO and Toluene in near the mixture with line presentation

Fig.S15.B Combining Asphaltene, DMSO and Toluene in near the mixture spherical presentation molecular model

Fig.S16

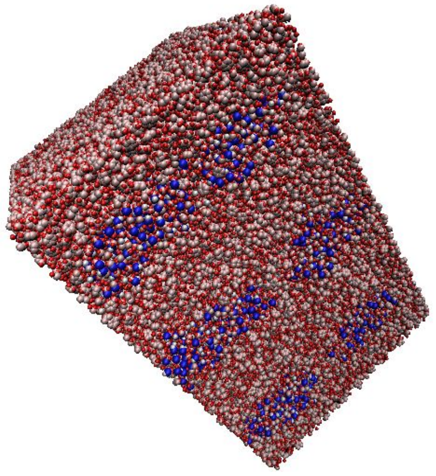

(A)

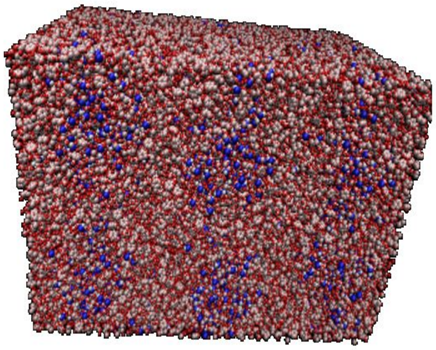

(B)

Fig.S16.A. **Cell size (144.45 * 144.45 * 216.68 A^3) Weight (5%Asphalt, 20 %DMSO, 75%Toluen),** Snapshot, cell size of mixture of Asphaltene with Toluene and DMSO with DFT potential parametrization at 0.8 ns

Fig.S16.B. **Cell Size (143.62*143.62*215.43 A^3)Weight (5%Asphalt, 20 %DMSO, 75%Toluen)** Snapshot of Asphaltene mixture in DMSO and Toluene at 1 ns

DFT result for MSD result for heavy aromatic core versus fractional time at different simulation time has been considered at Fig.S18. Based on DFT potential parametrization, low MSD value is observed at big simulation time.

Fig.S17

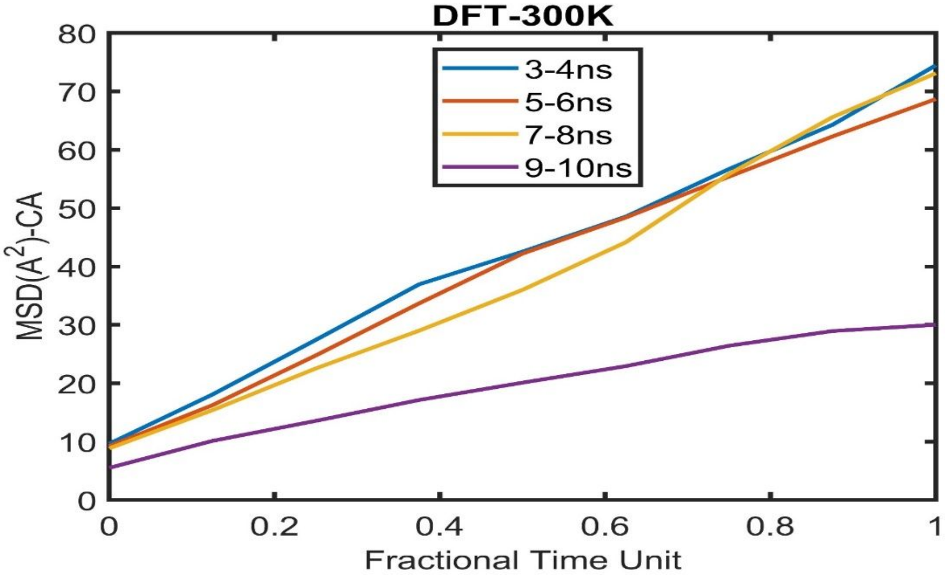

Fig.S17. MSD result for Asphaltene core from 3-10 nanosecond in different time interval versus fractional time unit based on DFT potential Parametrization

Fig.S18

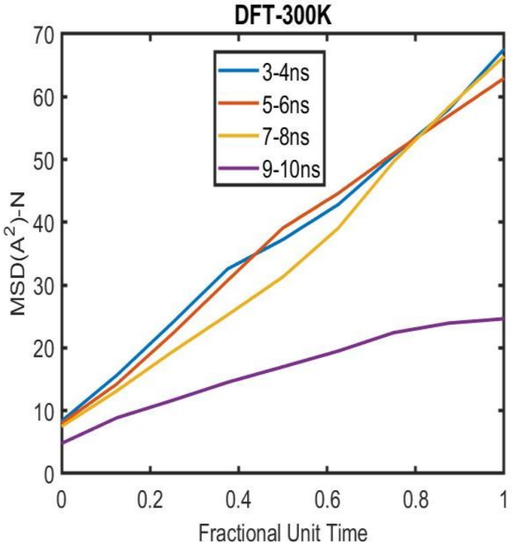

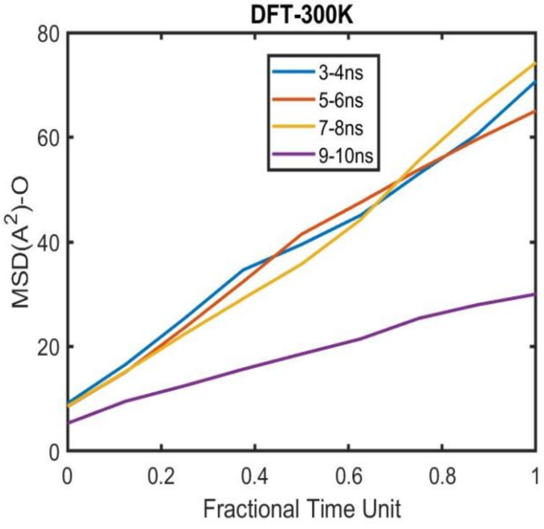

(A) **(B)**

Fig.S.18. A) Result of MSD for N atom in Asphaltene in during aggregation time based on DFT

FigS18. B) Result of MSD for O atom in Asphaltene in during aggregation time based on DFT

MSD result versus fractional time unit in different simulation time (ns; nanosecond) for core parts of heavy aromatic compound via new DFT developed potential for Nitrogen and oxygen atoms has been shown at Fig.S18.A and Fig.S18.B respectively.

Fig.S19

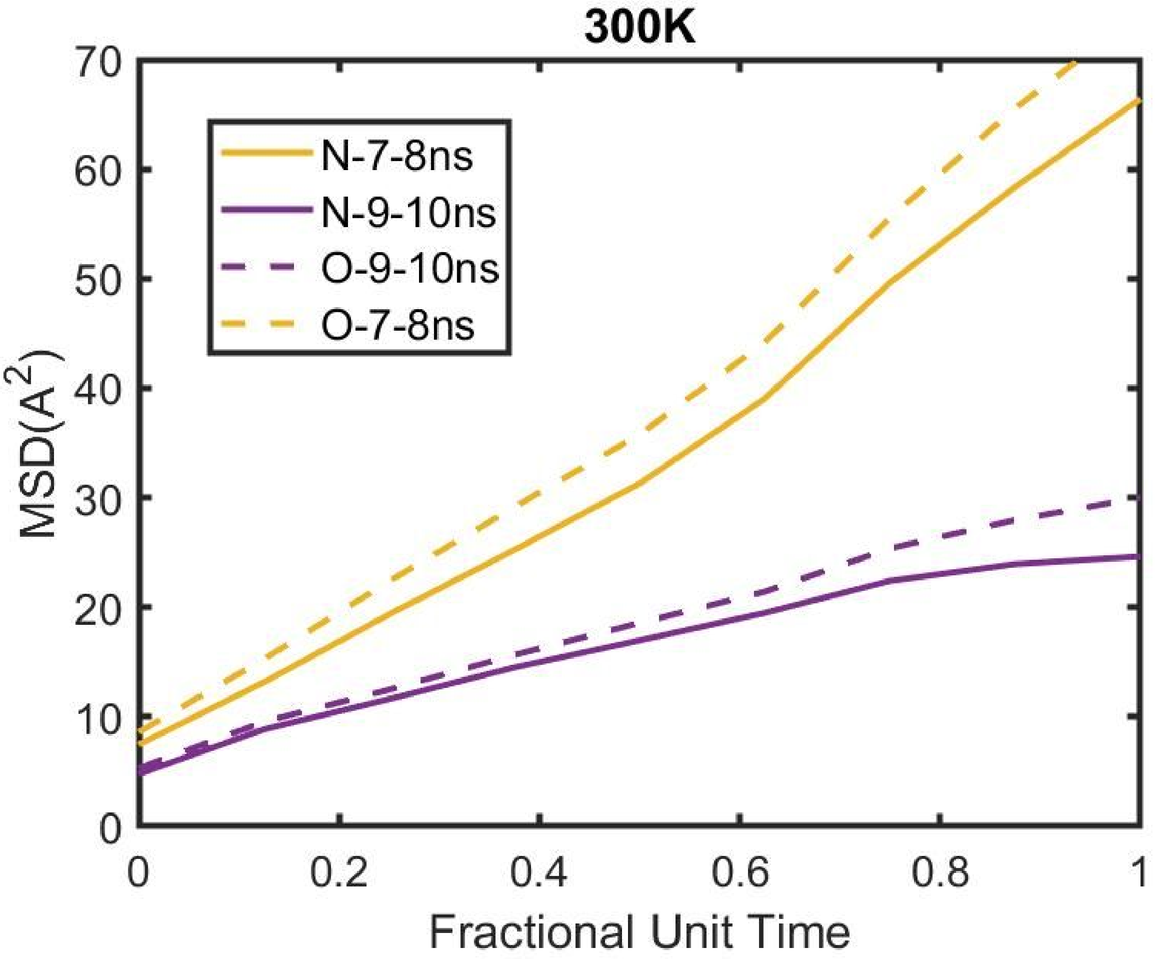

Fig.S19. Comparison MSD for N and O atoms in core parts of Asphalten

Comparison MSD for N and O atoms in core parts of pure heavy aromatic core has been presented at Fig.S19. Based on Fig.S19, two different regimes for diffusion of N and O atoms in heavy aromatic core is observed.

Fig.S20

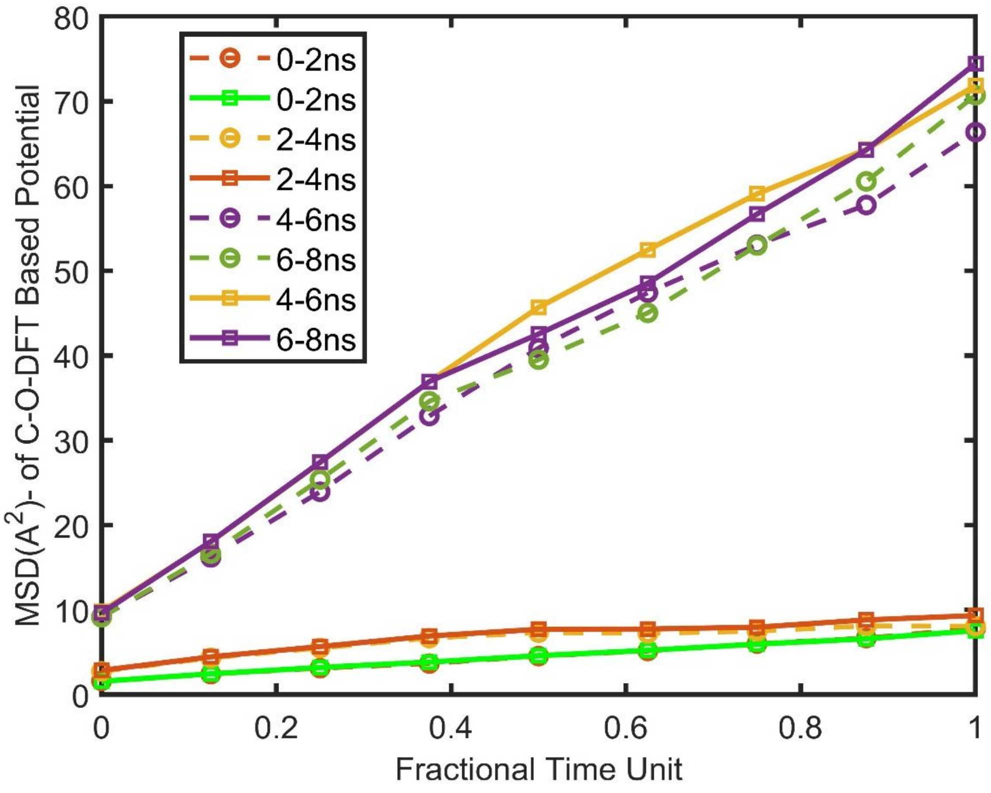

Fig.S20. MSD value for O- C in aromatic core, solid line for OPLS, dot line for DFT

Fig.S21

Asphalten Core

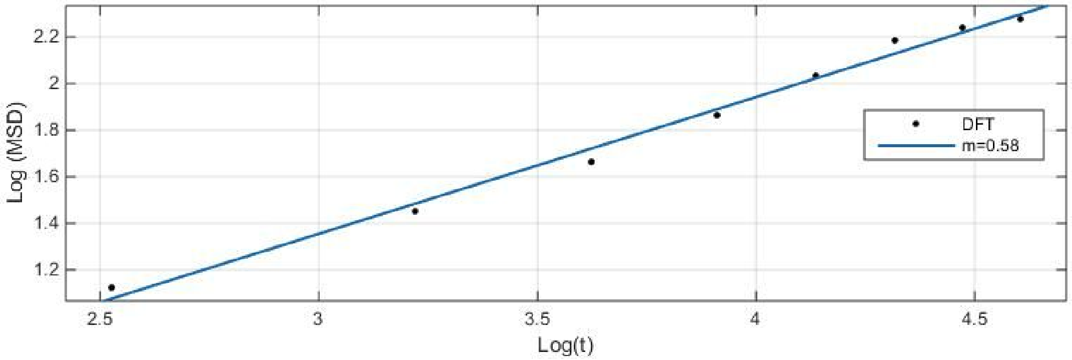

5-6 ns

Fig.S21. MSD versus time for asphalten core within mixture solvent DMSO-Toluene based on DFT calculation in time interval 5-6 ns

Fig.S22

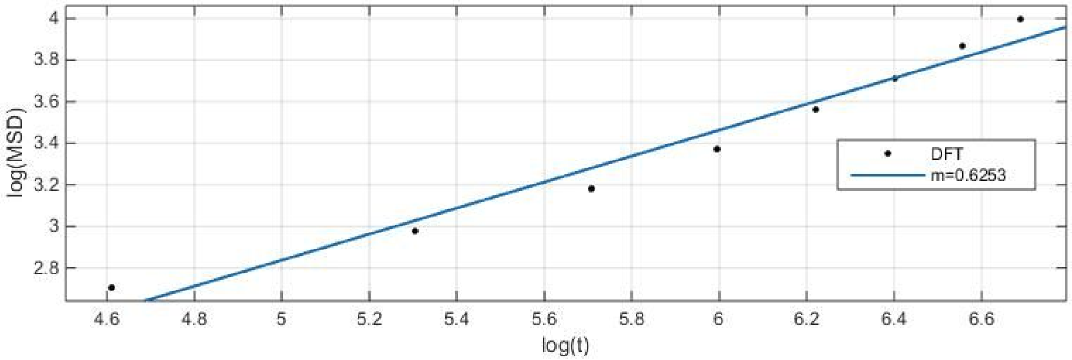

9-10ns

Fig.S22. MSD for asphalten core within mixture solvent DMSO-Toluene versus time in 9-10 ns

Fig.S23

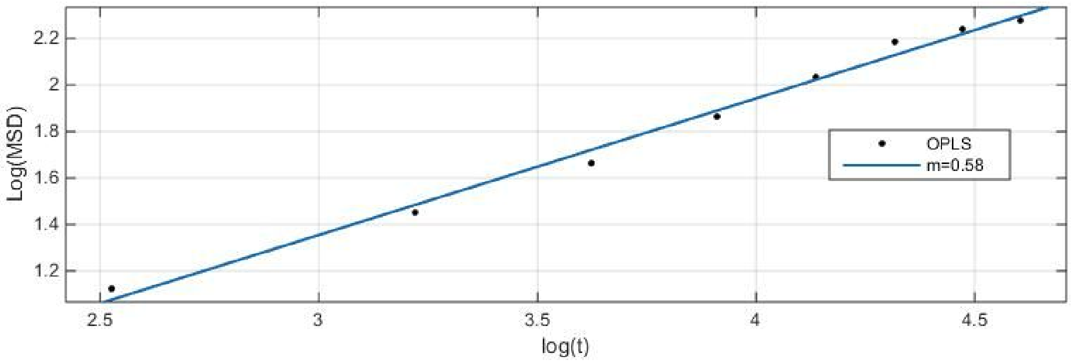

5-6 ns

Fig.S23. log MSD versus time for time interval 5-6 nanosecond for asphalten core within mixture solvent DMSO-Toluene via OPLS force field

Fig.S24

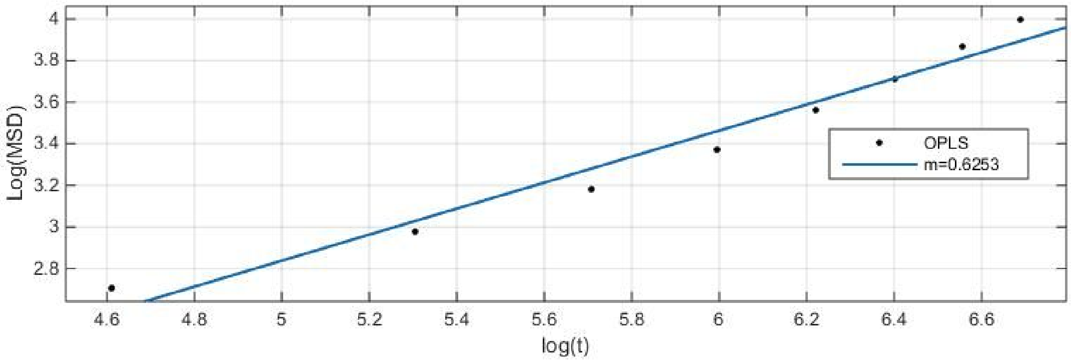

9-10ns

Fig.S24 log MSD versus time for time interval 9-10 ns nanosecond for asphalten core within mixture solvent DMSO-Toluene via OPLS force field

Also Log(MSD) versus Log(t) for asphalten core via OPLS force field at different simulation time (5-6) and (9-10) at Figure S24 and Fig.S25. According to the both Fig.S24 and Fig.S25, there is linear relationship for Log(MSD) versus Log(t)

Fig.S25

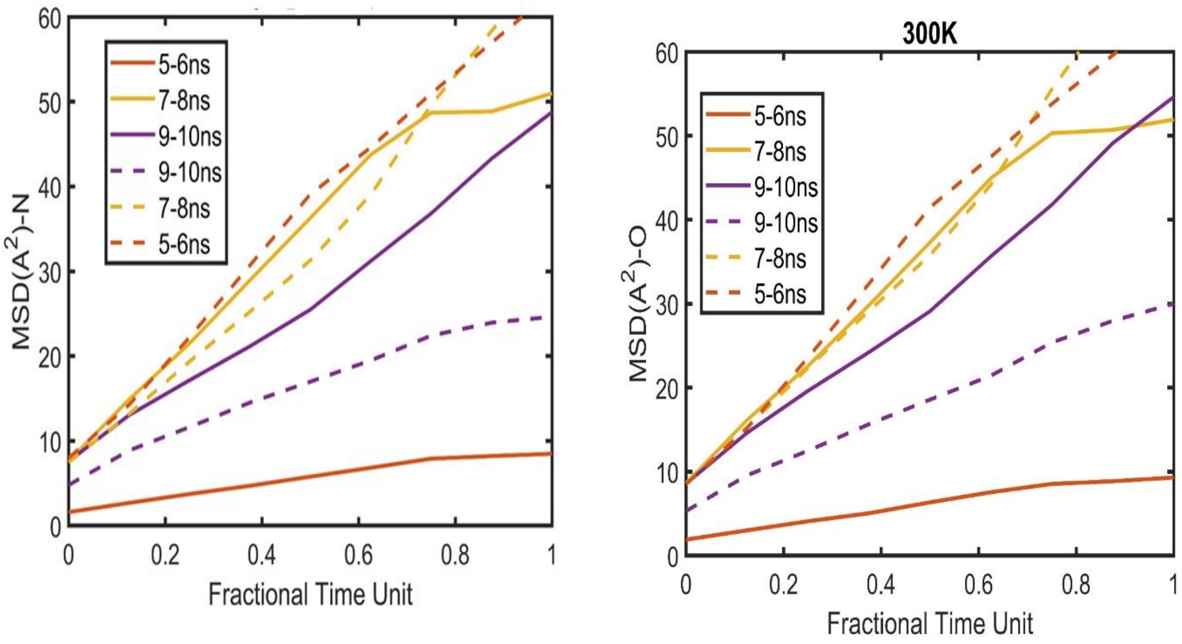

(A) (B)

Fig.S25.A.MSD result for nitrogen atom by using DFT potential parametrization and OPLS force field in mixture solvent, solid line for OPLS, dot line for DFT

Fig.S25.B. MSD result for O atom in core part of Asphaltene by application DFT potential parametrization and OPLS force field in mixture solvent, solid line for OPLS, dot line for DFT

MSD result versus fractional unit time in different simulation time for nitrogen and oxygen atoms as a core parts of heavy aromatic compound by using DFT potential parametrization and OPLS force field in mixture solvent has been presented at Fig.S25.A and Fig.S25.B respectively.

Fig.S26

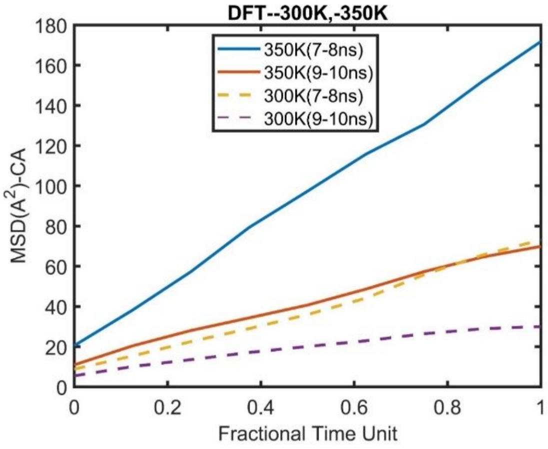

(A)

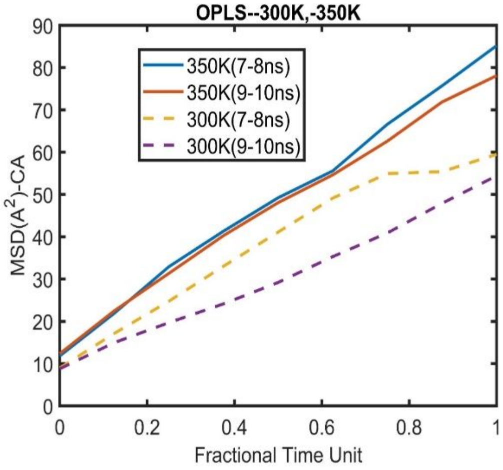

(B)

Fig.S26.A. MD result regarding temperature effect on MSD value by using DFT potential parametrization

Fig.S26.B.MSD value for Asphalten core by application OPLS force field at different temperature and time

Radial distribution function (RDF) between carbon-carbon in core parts of heavy aromatic CA-CA has been presented at Fig.S27 for 2ns and 10 ns simulation time. RDF shows that in during the simulation, radial distribution function does not change significantly. According to the Fig.S27, there is two main peaks for radial distribution function and also long range order is observed and they are not so much strong.

Fig.S27.

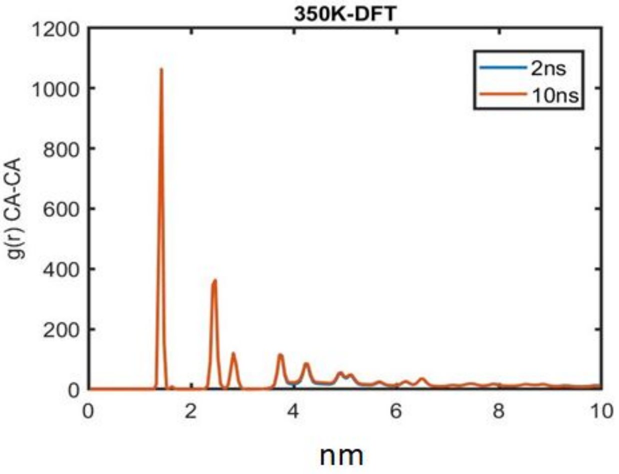

(1)

Fig.S27. Molecular dynamics result about radial distribution function based on DFT potential parametrization for carbon-carbon in core Asphaltene at 350K

Fig.S28

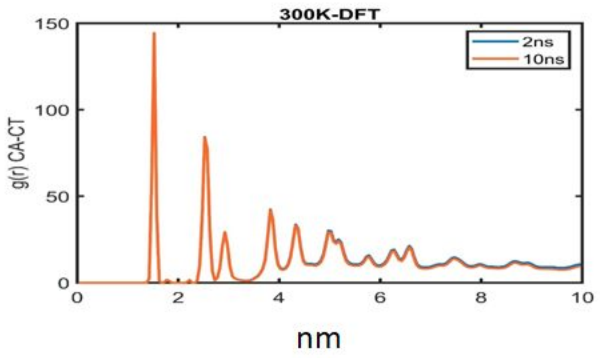

(2)

Fig.S28.Result of radial distribution function between carbon in aromatic core and aliphatic chain at two different simulation time 2, 10 ns

Chemical Symbol for Asphalten Structure

CA	12.0115000000000
CA	12.0115000000000
CA	12.0115000000000
CA	12.0115000000000
CA	12.0115000000000
CA	12.0115000000000
CA	12.0115000000000
CA	12.0115000000000
CA	12.0115000000000
HA	1.00797000000000
NB	14.0067000000000
CA	12.0115000000000
CA	12.0115000000000
CA	12.0115000000000
CA	12.0115000000000
CT	12.0115000000000
CT	12.0115000000000
CA	12.0115000000000
CA	12.0115000000000
HC	1.00797000000000
CT	12.0115000000000
HC	1.00797000000000
HC	1.00797000000000
CA	12.0115000000000
CA	12.0115000000000
CA	12.0115000000000
C56A	12.0115000000000
C56B	12.0115000000000
CA	12.0115000000000

OHP	15.9994000000000
HO	1.00797000000000
SA	32.0610000000000
C5A	12.0115000000000
C5B	12.0115000000000
HC	1.00797000000000
CT	12.0115000000000
HC	1.00797000000000
HC	1.00797000000000
CT	12.0115000000000
CT	12.0115000000000
HC	1.00797000000000
HC	1.00797000000000
CT	12.0115000000000
HC	1.00797000000000
HC	1.00797000000000
CT	12.0115000000000
HC	1.00797000000000
HC	1.00797000000000
HC	1.00797000000000
CT	12.0115000000000
CT	12.0115000000000
HC	1.00797000000000
HC	1.00797000000000
CT	12.0115000000000
HC	1.00797000000000
HC	1.00797000000000
CT	12.0115000000000
HC	1.00797000000000
HC	1.00797000000000
HC	1.00797000000000

HC	1.00797000000000
CT	12.0115000000000
CT	12.0115000000000
HC	1.00797000000000
HC	1.00797000000000
CT	12.0115000000000
HC	1.00797000000000
HC	1.00797000000000
HC	1.00797000000000
HC	1.00797000000000
HC	1.00797000000000
CT	12.0115000000000
HC	1.00797000000000
HC	1.00797000000000
HC	1.00797000000000
CA	12.0115000000000
CA	12.0115000000000
CA	12.0115000000000
CA	12.0115000000000
CA	12.0115000000000
CA	12.0115000000000
CA	12.0115000000000
CT	12.0115000000000
CA	12.0115000000000
CA	12.0115000000000
CA	12.0115000000000
CA	12.0115000000000
CA	12.0115000000000
CA	12.0115000000000
CA	12.0115000000000
CA	12.0115000000000

CT	12.0115000000000
HC	1.007970000000
HC	1.00797000000000
HC	1.00797000000000
CT	12.0115000000000
CT	12.0115000000000
CA	12.0115000000000
CA	12.0115000000000
CA	12.0115000000000
CA	12.0115000000000
CA	12.0115000000000
CA	12.0115000000000
CA	12.0115000000000
CT	12.0115000000000
CT	12.0115000000000
CT	12.0115000000000
CA	12.0115000000000
CA	12.0115000000000
CT	12.0115000000000
CT	12.0115000000000
CT	12.0115000000000
CT	12.0115000000000
CT	12.0115000000000
HC	1.00797000000000
HC	1.00797000000000
HC	1.00797000000000
HA	1.00797000000000
HC	1.00797000000000
HC	1.00797000000000
HC	1.00797000000000
HC	1.00797000000000

HC 1.00797000000000

HC 1.00797000000000

HC 1.00797000000000

HC 1.00797000000000

HC 1.00797000000000

HC 1.00797000000000

HC 1.00797000000000

HC 1.00797000000000

HC 1.00797000000000

HC 1.00797000000000

HA 1.00797000000000

HA 1.00797000000000

HC 1.00797000000000

HA 1.00797000000000

HC 1.00797000000000

HC 1.00797000000000

HC 1.00797000000000

HA 1.00797000000000

HC 1.00797000000000

HC 1.00797000000000

HC 1.00797000000000

HA 1.00797000000000

HA 1.00797000000000

CA , CT , HA, HC are aromatic carbon, aliphatic carbon, hydrogen which bonded aliphatic, hydrogen is bonded to aromatic in Asphalten respectively.

OHP, SA, NA, C56A, C56B, C5A, C5B, HO are oxygen atom, sulfur atom, nitrogen atom, carbon near the oxygen, carbon near the nitrogen, hydrogen bonded to oxygen in Asphalten respectively.

Lenard Jones parameters between solvent and heavy aromatics is presented as following

Lenard Jones Parameters in simulation, Energy UNITS kcal (epsilon) , sigma unit is Angstrom.

$$\sigma_{i,j} = (\sigma_{ii}\sigma_{jj})^{1/2} \qquad \varepsilon_{i,j} = (\varepsilon_{ii}\varepsilon_{jj})^{1/2}$$

VDW 210

CA	CA	lj	0.0700	3.5500
HA	CA	lj	0.0458	2.9310
NB	CA	lj	0.1091	3.3967
CT	CA	lj	0.0680	3.5249
HC	CA	lj	0.0458	2.9791
C56A	CA	lj	0.0700	3.5500
C56B	CA	lj	0.0700	3.5500
OHP	CA	lj	0.1091	3.3013
HO	CA	lj	0.0458	1.3323
SA	CA	lj	0.1663	3.5550
C5A	CA	lj	0.0700	3.5500
C5B	CA	lj	0.0700	3.5500
HA	HA	lj	0.0300	2.4200
NB	HA	lj	0.0714	2.8045
CT	HA	lj	0.0445	2.9103
HC	HA	lj	0.0300	2.4597
C56A	HA	lj	0.0458	2.9310
C56B	HA	lj	0.0458	2.9310
OHP	HA	lj	0.0714	2.7257
HO	HA	lj	0.0300	1.1000
SA	HA	lj	0.1089	2.9352
C5A	HA	lj	0.0458	2.9310
C5B	HA	lj	0.0458	2.9310
NB	NB	lj	0.1700	3.2500
CT	NB	lj	0.1059	3.3727
HC	NB	lj	0.0714	2.8504
C56A	NB	lj	0.1091	3.3967

C56B	NB	lj	0.1091	3.3967
OHP	NB	lj	0.1700	3.1587
HO	NB	lj	0.0714	1.2748
SA	NB	lj	0.2591	3.4015
C5A	NB	lj	0.1091	3.3967
C5B	NB	lj	0.1091	3.3967
CT	CT	lj	0.0660	3.5000
HC	CT	lj	0.0445	2.9580
C56A	CT	lj	0.0680	3.5249
C56B	CT	lj	0.0680	3.5249
OHP	CT	lj	0.1059	3.2780
HO	CT	lj	0.0445	1.3229
SA	CT	lj	0.1615	3.5299
C5A	CT	lj	0.0680	3.5249
C5B	CT	lj	0.0680	3.5249
HC	HC	lj	0.0300	2.5000
C56A	HC	lj	0.0458	2.9791
C56B	HC	lj	0.0458	2.9791
OHP	HC	lj	0.0714	2.7704
HO	HC	lj	0.0300	1.1180
SA	HC	lj	0.1089	2.9833
C5A	HC	lj	0.0458	2.9791
C5B	HC	lj	0.0458	2.9791
C56A	C56A	lj	0.0700	3.5500
C56B	C56A	lj	0.0700	3.5500
OHP	C56A	lj	0.1091	3.3013
HO	C56A	lj	0.0458	1.3323
SA	C56A	lj	0.1663	3.5550
C5A	C56A	lj	0.0700	3.5500
C5B	C56A	lj	0.0700	3.5500
C56B	C56B	lj	0.0700	3.5500

OHP	C56B	lj	0.1091	3.3013
HO	C56B	lj	0.0458	1.3323
SA	C56B	lj	0.1663	3.5550
C5A	C56B	lj	0.0700	3.5500
C5B	C56B	lj	0.0700	3.5500
OHP	OHP	lj	0.1700	3.0700
HO	OHP	lj	0.0714	1.2390
SA	OHP	lj	0.2591	3.3059
C5A	OHP	lj	0.1091	3.3013
C5B	OHP	lj	0.1091	3.3013
HO	HO	lj	0.0300	0.5000
SA	HO	lj	0.1089	1.3342
C5A	HO	lj	0.0458	1.3323
C5B	HO	lj	0.0458	1.3323
SA	SA	lj	0.3950	3.5600
C5A	SA	lj	0.1663	3.5550
C5B	SA	lj	0.1663	3.5550
C5A	C5A	lj	0.0700	3.5500
C5B	C5A	lj	0.0700	3.5500
C5B	C5B	lj	0.0700	3.5500
CA	SZds	lj	0.166282	3.5500
CA	OZds	lj	0.14	3.120
CA	CTds	lj	0.06797	3.52
CA	HCds	lj	0.04582	3.02
HA	SZds	lj	0.1088	2.99
HA	OZds	lj	0.09165	2.55
HA	CTds	lj	0.044497	2.96
HA	HCds	lj	0.03	2.46
NB	SZds	lj	0.2591	3.405
NB	OZds	lj	0.2181	2.975
NB	CTds	lj	0.1059	3.375

NB	HCds	lj	0.07141	2.875
CT	SZds	lj	0.16146	3.53
CT	OZds	lj	0.1359	3.10
CT	CTds	lj	0.066	3.50
CT	HCds	lj	0.04449	3.00
HC	SZds	lj	0.1088	3.03
HC	OZds	lj	0.09165	2.60
HC	CTds	lj	0.04449	3.0
HC	HCds	lj	0.03	2.5
C56A	SZds	lj	0.1662	3.555
C56A	OZds	lj	0.14	3.125
C56A	CTds	lj	0.06797	3.525
C56A	HCds	lj	0.04582	3.025
C56B	SZds	lj	0.1662	3.555
C56B	OZds	lj	0.14	3.125
C56B	CTds	lj	0.067970	3.525
C56B	HCds	lj	0.04582	3.025
OHP	SZds	lj	0.25913	3.315
OHP	OZds	lj	0.21817	2.885
OHP	CTds	lj	0.10592	3.285
OHP	HCds	lj	0.071414	2.785
HO	SZds	lj	0.10885	2.03
HO	OZds	lj	0.09165	1.60
HO	CTds	lj	0.04449	2.0
HO	HCds	lj	0.0300	1.5
SA	SZds	lj	0.395	3.56
SA	OZds	lj	0.3325	3.13
SA	CTds	lj	0.1614	3.53
SA	HCds	lj	0.1088	3.03
C5A	SZds	lj	0.16628	3.555
C5A	OZds	lj	0.14	3.125

C5A	CTds	lj	0.06797	3.525
C5A	HCds	lj	0.04582	3.025
C5B	SZds	lj	0.16628	3.555
C5B	OZds	lj	0.14	3.125
C5B	CTds	lj	0.06797	3.525
C5B	HCds	lj	0.04582	3.025
SZds	SZds	lj	0.3950	3.5600
OZds	SZds	lj	0.3326	3.1003
CTds	SZds	lj	0.1615	3.5299
HCds	SZds	lj	0.1089	2.9833
OZds	OZds	lj	0.2800	2.7000
CTds	OZds	lj	0.1359	3.0741
HCds	OZds	lj	0.0917	2.5981
CTds	CTds	lj	0.0660	3.5000
HCds	CTds	lj	0.0445	2.9580
HCds	HCds	lj	0.0300	2.5000
CA	CAts	lj	0.07	3.55
CA	HAts	lj	0.04582	2.985
CA	CTts	lj	0.06797	3.525
CA	HCts	lj	0.045825	3.025
HA	CAts	lj	0.045825	2.985
HA	HAts	lj	0.03	2.42
HA	CTts	lj	0.04449	2.96
HA	HCts	lj	0.03	2.46
NB	CAts	lj	0.1090	3.4
NB	HAts	lj	0.07141	2.835
NB	CTts	lj	0.10592	3.375
NB	HCts	lj	0.07141	2.875
CT	CAts	lj	0.06797	3.525
CT	HAts	lj	0.04449	2.96
CT	CTts	lj	0.066	3.5

CT	HCts	lj	0.04449	3.00
HC	CAts	lj	0.04582	3.025
HC	HAts	lj	0.03	2.46
HC	CTts	lj	0.04449	3.00
HC	HCts	lj	0.03	2.5
C56A	CAts	lj	0.07	3.55
C56A	HAts	lj	0.04582	2.985
C56A	CTts	lj	0.06797	3.525
C56A	HCts	lj	0.04582	3.025
C56B	CAts	lj	0.07	3.55
C56B	HAts	lj	0.04582	2.985
C56B	CTts	lj	0.06797	3.525
C56B	HCts	lj	0.04582	3.025
OHP	CAts	lj	0.1090	3.54
OHP	HAts	lj	0.07141	2.745
OHP	CTts	lj	0.1059	3.285
OHP	HCts	lj	0.07141	2.785
HO	CAts	lj	0.04582	2.025
HO	HAts	lj	0.03	1.46
HO	CTts	lj	0.04449	2
HO	HCts	lj	0.03	1.5
SA	CAts	lj	0.16628	3.555
SA	HAts	lj	0.10885	2.99
SA	CTts	lj	0.16146	3.53
SA	HCts	lj	0.10885	3.03
C5A	CAts	lj	0.07	3.55
C5A	HAts	lj	0.04582	2.985
C5A	CTts	lj	0.06797	3.525
C5A	HCts	lj	0.04582	3.025
C5B	CAts	lj	0.07	3.55
C5B	HAts	lj	0.04582	2.985

C5B	CTts	lj	0.06797	3.525
C5B	HCts	lj	0.04582	3.025
CAts	SZds	lj	0.1662	3.555
CAts	OZds	lj	0.14	3.125
CAts	CTds	lj	0.06797	3.525
CAts	HCds	lj	0.04582	3.025
HAts	SZds	lj	0.10885	2.99
HAts	OZds	lj	0.09165	2.56
HAts	CTds	lj	0.04449	2.96
HAts	HCds	lj	0.03	2.46
CTts	SZds	lj	0.1614	3.53
CTts	OZds	lj	0.1359	3.10
CTts	CTds	lj	0.066	3.5
CTts	HCds	lj	0.04449	3.00
HCts	SZds	lj	0.10885	3.03
HCts	OZds	lj	0.09165	2.60
HCts	CTds	lj	0.04449	3.00
HCts	HCds	lj	0.03	2.50
CAts	CAts	lj	0.0700	3.5500
HAts	CAts	lj	0.0458	2.9310
CTts	CAts	lj	0.0680	3.5249
HCts	CAts	lj	0.0458	2.9791
HAts	HAts	lj	0.0300	2.4200
CTts	HAts	lj	0.0445	2.9103
HCts	HAts	lj	0.0300	2.4597
CTts	CTts	lj	0.0660	3.5000
HCts	CTts	lj	0.0445	2.9580
HCts	HCts	lj	0.0300	2.5000

There are 210 types of Lenard's jones for Asphalten which is solved in Toluene and DMSO solvents. ts symbols has been used to represent atoms of toluene solvent (ts) molecule. "ds" symbols presents for DMSO solvent atoms.
